# Machine learning models for early sepsis recognition in the neonatal intensive care unit using readily available electronic health record data

**DOI:** 10.1371/journal.pone.0212665

**Published:** 2019-02-22

**Authors:** Aaron J. Masino, Mary Catherine Harris, Daniel Forsyth, Svetlana Ostapenko, Lakshmi Srinivasan, Christopher P. Bonafide, Fran Balamuth, Melissa Schmatz, Robert W. Grundmeier

**Affiliations:** 1 Department of Anesthesiology and Critical Care, Perelman School of Medicine at the University of Pennsylvania, Philadelphia, Pennsylvania, United States of America; 2 Department of Biomedical and Health Informatics, Children’s Hospital of Philadelphia, Philadelphia, Pennsylvania, United States of America; 3 Department of Pediatrics, Perelman School of Medicine at the University of Pennsylvania, Philadelphia, Pennsylvania, United States of America; 4 Department of Pediatrics, Children’s Hospital of Philadelphia, Philadelphia, PA, United States of America; 5 Center for Pediatric Clinical Effectiveness, Children’s Hospital of Philadelphia, Philadelphia, PA, United States of America; University of Murcia, SPAIN

## Abstract

**Background:**

Rapid antibiotic administration is known to improve sepsis outcomes, however early diagnosis remains challenging due to complex presentation. Our objective was to develop a model using readily available electronic health record (EHR) data capable of recognizing infant sepsis at least 4 hours prior to clinical recognition.

**Methods and findings:**

We performed a retrospective case control study of infants hospitalized ≥48 hours in the Neonatal Intensive Care Unit (NICU) at the Children’s Hospital of Philadelphia between September 2014 and November 2017 who received at least one sepsis evaluation before 12 months of age. We considered two evaluation outcomes as cases: *culture positive*–positive blood culture for a known pathogen (110 evaluations); and *clinically positive*–negative cultures but antibiotics administered for ≥120 hours (265 evaluations). Case data was taken from the 44-hour window ending 4 hours prior to evaluation. We randomly sampled 1,100 44-hour windows of control data from all times ≥10 days removed from any evaluation. Model inputs consisted of up to 36 features derived from routine EHR data. Using 10-fold nested cross-validation, 8 machine learning models were trained to classify inputs as sepsis positive or negative. When tasked with discriminating culture positive cases from controls, 6 models achieved a mean area under the receiver operating characteristic (AUC) between 0.80–0.82 with no significant differences between them. Including both culture and clinically positive cases, the same 6 models achieved an AUC between 0.85–0.87, again with no significant differences.

**Conclusions:**

Machine learning models can identify infants with sepsis in the NICU hours prior to clinical recognition. Learning curves indicate model improvement may be achieved with additional training examples. Additional input features may also improve performance. Further research is warranted to assess potential performance improvements and clinical efficacy in a prospective trial.

## Introduction

Sepsis is a major cause of morbidity and mortality in infants worldwide [1,2]. Although sepsis affects relatively few healthy, term infants, the incidence is 200-fold higher in those born prematurely or chronically hospitalized [3,4]. More than 50% of extremely preterm infants will have an evaluation for invasive infection and one third will develop sepsis during their NICU stay [5]. Prematurely born infants experience the highest mortality (7–28%), and among survivors, 30–50% incur major long-term impairments including prolonged hospitalization, chronic lung disease and neurodevelopmental disabilities [6–8]. Importantly, recent data highlight the exponential rise of associated healthcare costs and burdens faced not only by sepsis survivors but also by their caregivers [9]. To date, despite increased understanding of the pathophysiology of sepsis and sophistication of neonatal intensive care strategies, including clinical decision support efforts, there have been only modest improvements in outcomes from sepsis in infants [10].

Following bacterial invasion of the bloodstream, the immune system initiates a potentially damaging systemic inflammatory response syndrome (SIRS) that may quickly progress to severe sepsis, multi-organ system failure and death [9,11]. Early sepsis recognition, therefore, followed by timely intervention, is key to reducing morbidity and mortality. Recent studies address the consequences of delayed treatment of infected adults and children [12,13]. In a study of 49,331 adults from the New York State Department of Health, delays in time to antibiotics were associated with significantly increased risk-adjusted odds of mortality (1.04 per hour) [13]. Delayed antimicrobial therapy was shown to be an independent risk factor for prolonged organ dysfunction and mortality in a study of 130 critically ill children [12]. Despite the importance of early intervention, delays in recognition and treatment are common [14]. Infants frequently demonstrate subtle, ambiguous clinical signs, which mimic other neonatal disease processes. Screening laboratory tests have limited diagnostic accuracy in neonatal sepsis, making rapid diagnosis difficult. The blood culture, the reference standard for sepsis diagnosis, may be falsely negative due to the small volume of blood obtained as well as the low density of infecting microorganisms [15–17]. Consequently, infants suspected to have sepsis despite negative cultures, are often managed conservatively and receive prolonged antibiotic therapy.

Machine learning and statistical modeling approaches have been applied in previous studies in an effort to address the challenges associated with sepsis recognition and care management [18–32]. Several studies used machine learning models to identify individuals most at risk for sepsis related mortality [19,25,26]. Statistical models can predict septic shock as much as 28 hours before onset [18]. While such models may inform clinical decision support tools that lead to care adjustment for patients with confirmed sepsis, they are less likely to support early recognition.

Other studies developed machine learning models to confirm clinician suspected sepsis. A study of 299 infants compared the ability of several machine learning algorithms to confirm suspected sepsis using data available up to 12 hours after clinical recognition but before blood culture results were available [29]. More recent efforts have augmented standard EHR data with features extracted from admission notes using natural language processing (NLP) to confirm suspected sepsis in adults [30]. Although such models may reduce time to confirmation as compared to laboratory tests, they are unlikely to substantially reduce time to antibiotic treatment, which is typically initiated at the time of blood culture.

Some studies have sought to develop models that identify sepsis incidence prior to clinician suspicion and thereby enable earlier treatment. In infants, a statistical prediction model (HeRO score) reduced sepsis related mortality in very low birth weight infants (<1500 grams), presumably by supporting earlier recognition [27,28]. However, in a subsequent, large retrospective study, the HeRO score failed to detect neonatal sepsis, suggesting the predictive value is uncertain in clinical practice [33]. Additional studies used a novel network representation of vital sign dynamics [22,23] for sepsis prediction in adults. However, these models require input features derived from heart rate measurements collected every few seconds from bedside monitors, which are not typically available in most EHRs thereby limiting their general applicability. Two recent studies used hourly vital sign and demographic variables in a proprietary algorithm (InSight) to predict adult sepsis 4 hours prior to clinician suspicion [31]. Although these works utilize similar methods, significant physiological and immunological differences between adults and children may preclude direct application to infants.

Our objective was to develop and evaluate a machine learning model specifically designed to recognize sepsis in infants hospitalized in the neonatal intensive care unit at least 4 hours prior to clinical suspicion. Unlike the previous infant study, we developed our sepsis prediction models using only readily available EHR data in an effort to enhance general applicability. To our knowledge, this is one of only a limited number of studies to investigate machine learning for sepsis identification prior to clinical recognition, and the first for infant sepsis identification prior to clinical recognition using only routinely collected EHR data.

In the following sections, we describe the development of 8 machine learning models tasked with differentiating patient data collected 4 hours prior to clinical suspicion of sepsis, as indicated by time of draw for culture, from patient data collected during periods with no evidence of sepsis. We describe our training and evaluation datasets—consisting of information routinely available in most EHRs—for a population of infants hospitalized in the Children’s Hospital of Philadelphia (CHOP) neonatal intensive care unit (NICU). Finally, we present an evaluation of model performance demonstrating that several of the presented models are able to recognize infant sepsis 4 hours prior to clinical suspicion at least as well as those reported in prior adult studies.

## Materials and methods

**Fig 1:**
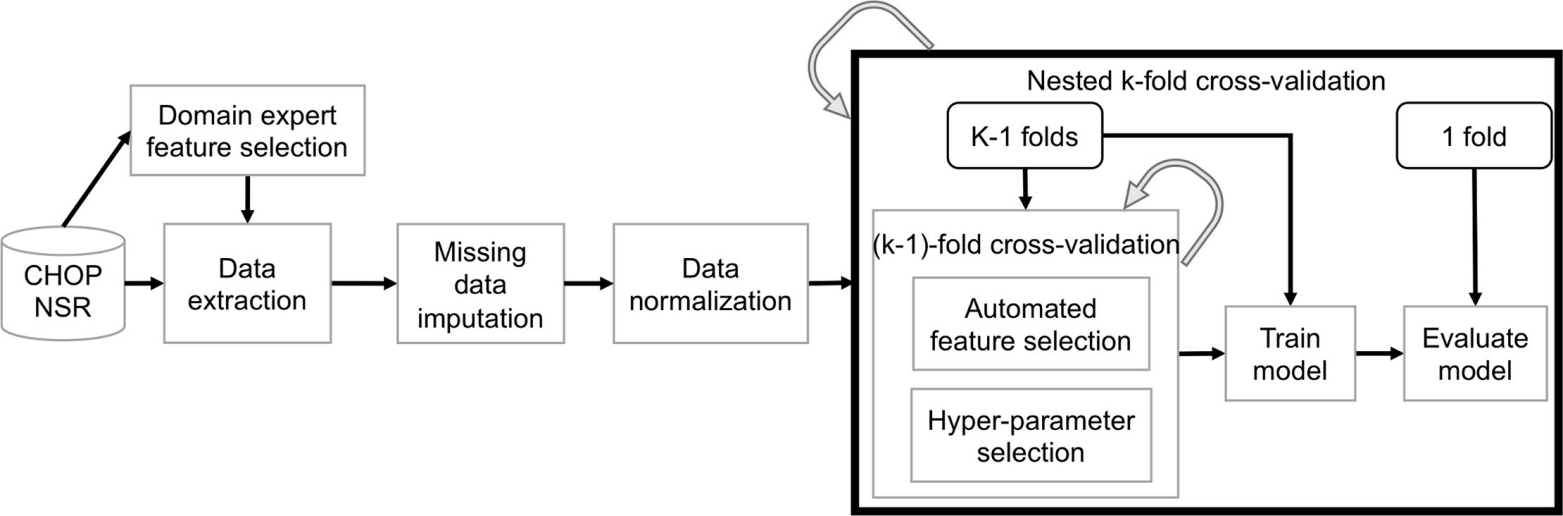
Experimental workflow. Data was obtained from the CHOP NICU Sepsis Registry (NSR). Domain expert review was used to identify an initial feature set. Continuous data was normalized. Mean data imputation was used to complete missing data. Nested k-fold cross-validation, in which the complete dataset is divided into *k* stratified bins of approximately equal size (*k = 10* in our study), was used to train and evaluate models. The curved arrows indicate loops over the data folds. The outer loop runs over all *k* folds. For each iteration, a fold is reserved for testing. The remaining *k-1* folds are passed to the inner loop, which performs standard *k-fold cross-validation* to automatically select features and model tuning parameters. Mutual information between individual features and target class was used for automated feature selection. The model is then trained using *k-1* folds and evaluated on the held-out fold. This process is repeated *k* times so that each data fold is used once for evaluation.

All data in this study was extracted automatically from the EHR and anonymized prior to transfer to the study database. Study data did include dates of service and dates of birth, however there were no direct patient identifiers such as medical record numbers or patient names in the dataset. The Institutional Review Board at the Children’s Hospital of Philadelphia approved this research study and waived the requirement for consent. The experimental workflow for this study is illustrated in Fig 1.

### Study design and setting

We implemented a retrospective case control study among infants hospitalized in the NICU at CHOP in which individuals were allowed to serve as their own controls. The CHOP NICU is a 99-bed quaternary unit that admits and treats roughly 1,100 medically and surgically complex infants annually.

### Study subjects

We included infants hospitalized for at least 48 hours in the NICU at the Children’s Hospital of Philadelphia (CHOP) between September 2014 and November 2017 who received at least one evaluation for sepsis before 12 months of age. We excluded sepsis evaluations that were performed within 48 hours of admission to the CHOP NICU due to lack of sufficient baseline observation time. This cohort of infants contributed both case and control periods of time in the analysis as outlined in the sections below.

We considered two possible outcomes from sepsis evaluations as cases in our analyses: *culture positive* if the evaluation yielded a positive blood culture for a pathogen; and *clinically positive* if cultures were negative, but antibiotics were administered for at least 120 hours. Other possible outcomes from sepsis evaluations, which were excluded as cases, were: (1) negative cultures and less than 72 hours of antibiotic administration (i.e. not sepsis); (2) cultures positive for bacteria from sources other than blood; (3) positive cultures for viral or fungal pathogens; and (4) indeterminate results due to pending cultures at the time of data extraction, cultures positive for known contaminants (i.e. non-pathogenic bacteria), or administration of antibiotics for at least 72 hours but less than 120 hours (i.e. uncertainty about whether the infant had clinical sepsis). Although these infants may have physiologic changes that overlap with those of infants experiencing bacterial bloodstream infections, we excluded observation times around these evaluations from consideration since disease processes other than culture-proven or clinically-presumed bacterial bloodstream infections were not the focus of our study.

### Identification of case periods

There is no literature that has defined the transition time from an infant’s normal health state to a state of critical illness associated with sepsis onset. However, prior research suggests that an incubation period of two days is sufficient for detection of bacteria in blood culture [34,35]. Consequently, it seems plausible that there may be physiologic changes due to sepsis 48 hours prior to clinical presentation but unlikely that there would be detectable changes more than 48 hours prior. As our goal was to predict sepsis 4 hours prior to current clinical recognition, we obtained case data from the 44-hour window starting 48 hours prior to clinical evaluation (T_-48h_) and ending 4 hours prior to evaluation (T_-4h_).

### Identification of control periods

**Fig 2:**
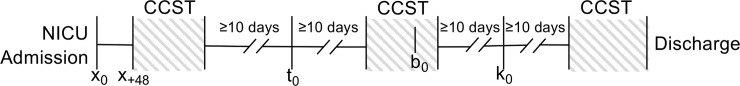
Timeline representation of a hypothetical NICU hospitalization and corresponding sepsis data sampling scheme. Sepsis evaluation times are indicated by t_0_ and k_0_. For this hypothetical scenario, case data is taken from the two 44-hour windows, [t_-48_, t_-4_] and [k_-48_, k_-4_], starting 4 hours prior to blood draw, t_0_ and k_0_, for the two sepsis evaluations. Time indices, t_-n_, indicate times *n* hours prior to blood draw. In this scenario, individual control start times are randomly selected from all candidate control start times (CCST) (indicated by the shaded regions). CCST include all times starting on day 3 after admission (indicated by x_0_) that are separated by at least 10 days from any sepsis evaluation time. For a randomly selected control start time, b_0_, control data is taken from the 44-hour window [b_-48_, b_-4_].

As illustrated in Fig 2, we identified candidate control start times as any time point during an included individual’s hospitalization in the NICU beginning 48 hours after admission for which there were no sepsis evaluations within 10 days. Note, these criteria allow for inclusion of candidate control times starting on day 3 of an individual’s NICU stay provided the individual did not receive a sepsis evaluation for at least ten days following the candidate control time. Since previously undetected sepsis is occasionally noted at autopsy for deceased infants, we similarly excluded the 10 days prior to death from consideration for possible control observations. Finally, since antibiotics are occasionally administered outside the immediate context of sepsis evaluations (e.g. unusually prolonged treatment for severe sepsis, or for certain surgical procedures), we also excluded observation time when systemic antibiotics typically used to treat sepsis were administered within the preceding 48 hours since it is less likely that an infant on such antibiotics will develop sepsis or demonstrate a positive blood culture. We then used random sampling with replacement of all available candidate control times to achieve datasets with a 9% and 25% incidence of culture positive and clinically positive cases, respectively, to reflect sepsis incidence rates similar to clinical observation. Since sepsis evaluations are not evenly distributed across all times of day, we weighted time of day in the sampling strategy to ensure a similar distribution between both case and control observations. We treated data in the 44-hour window ending four hours prior to each selected control time as a control sample.

### Data source

**Fig 3:**
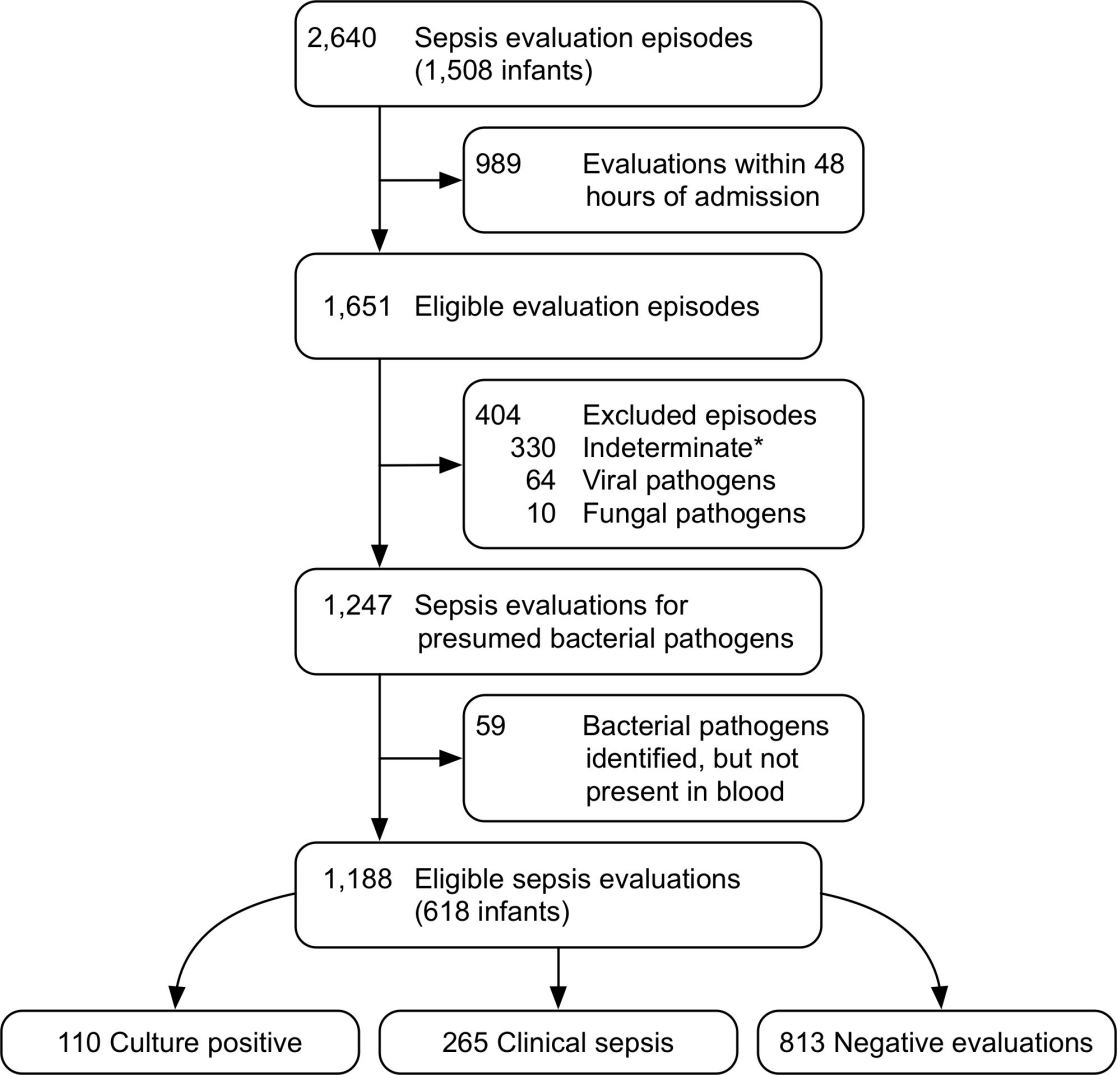
Study flow diagram. Excluded episodes, Indeterminate*: episodes with pending cultures at the time of data extraction, results that most likely represented contaminants, episodes with bacteria isolated from sources other than blood.

Data for this study was obtained from the CHOP NICU sepsis registry established in 2014. The registry is automatically populated with data abstracted from the EHR (Epic Systems, Inc., Verona, WI) for all infants evaluated for sepsis while hospitalized in the CHOP NICU. For each infant with at least one sepsis evaluation, the registry captures EHR data for a pre-determined list of variables including demographics, longitudinal vital signs (collected hourly), diagnosis, antibiotic, microbiological, and treatment data throughout the infant’s entire hospitalization. From this data source, we identified 618 unique infants with 1,188 sepsis evaluations that met the inclusion and exclusion criteria (see Fig 3). Demographic information is given in Table 1. There were 110 *culture positive* and 265 *clinically positive* evaluations included as cases.

**Table 1:** Demographics at time of initial sepsis evaluation. Columns 2–4 indicate values by evaluation result (individuals may have multiple evaluations). Last column indicates overall study population values. Values in brackets indicate number of individuals.

	Culture Positive	Clinical Sepsis	Negative	Study Population
**Number of Infants**	92	199	492	618
**Gestational Age (weeks)**	Median (32) Range (22, 40)	Median (32) Range (23, 41)	Median (34) Range (22, 41)	Median (34) Range (22, 41)
**Race (Percent)**				
White	39% [36]	39% [78]	43% [212]	43% [266]
Unknown	35% [32]	29% [59]	30% [148]	30% [184]
Black	23% [21]	23% [46]	22% [108]	21% [131]
Two or more	1% [1]	4% [7]	3% [13]	3% [19]
Asian	1% [1]	4% [7]	2% [10]	3% [16]
Native American/Alaskan Native	1% [1]	1% [2]	<1% [1]	<1% [2]
**Ethnicity (Percent)**				
Non-Hispanic	86% [79]	87% [174]	88% [435]	88% [542]
Hispanic	14% [13]	13% [25]	12% [57]	12% [76]
Unknown	0% []	0% []	0% []	0% []
**Age Days**	Median (25) Range (2, 235)	Median (23) Range (2, 164)	Median (16) Range (2, 322)	Median (17) Range (2, 322)
**Comorbidities**				
IVH or VP Shunt	7% [6]	10% [20]	8% [39]	8% [47]
Surgical Conditions	15% [14]	18% [36]	18% [89]	17% [104]
Congenital Heart Disease	11% [10]	8% [16]	9% [43]	8% [51]
Chronic Lung Disease	18% [17]	16% [32]	15% [76]	14% [87]
NEC	30% [28]	37% [74]	14% [70]	17% [107]

### Machine learning analysis

#### Feature selection and data imputation

Through literature and physician expert review, we identified 30 features collected in the NICU sepsis registry that are known or suspected to be associated with infant sepsis (see S1 Table) [36,37]. We derived 6 additional features from hourly vital signs including thresholds for temperature and fraction of inspired oxygen (FiO2), and the difference between the most recent measurement and the average over the previous 24 hours for heart rate, temperature, respiratory rate, and mean arterial blood pressure. The temperature threshold variable was set to 1 if temperature was less than 36 or greater than 38 degrees Celsius and 0 otherwise. The FiO2 threshold variable was intended as a surrogate measure to indicate high levels of ventilator support and was set to 1 if FiO2 was greater than or equal to 40% and 0 otherwise. Additional features included nursing assessments of clinical status (apnea, bradycardia, or desaturation events; lethargy; poor perfusion), indwelling lines (central venous line, umbilical artery catheter), and support (extracorporeal membrane oxygenation, mechanical ventilation). One area in which there is some overlap in factors contributing to increased risk for sepsis in both children and adults is the presence of underlying chronic medical conditions, perhaps related to immune dysfunction and impaired resistance to bacterial pathogens [38–40]. Given the shared increased risk observed in both populations, it seems plausible that the baseline risk of sepsis may also be higher among infants who have experienced co-morbid conditions such as necrotizing enterocolitis, prolonged ventilation for chronic lung disease, or surgical procedures (e.g. ventriculo-peritoneal shunt placement, cardiac procedures, or gastrointestinal surgeries). We therefore included indicator variables for the presence of these comorbidities in our analyses.

Prior to model selection and training, we calculated the percent of sepsis evaluations for which data was missing for each feature, see Table 2. Threshold features are not listed, as the percent missing is identical to that of the corresponding raw feature. Features that indicated the presence of co-morbid conditions, nursing assessments of clinical status, indwelling lines, and support are also not listed as they are considered as having no missing values. One variable, capillary pH, was missing for more than 80% of case evaluations and more than 90% of control samples and was therefore removed from the dataset. For the remaining features, we used mean imputation to replace missing values with the population mean calculated from the entire dataset. There are a number of imputation methods (e.g. KNN, MICE) that often perform better than mean imputation [41–43], however these methods typically introduce additional model parameters (e.g. KNN requires selection of a distance measure, the number of neighbors, and the weighting scheme used to compute the imputed value from the neighbor’s values) that increase the potential for variance (overfitting). Due to the constraint of our limited training dataset size, we sought to mitigate this concern by using mean imputation which does not require selection of any parameters. Finally, post imputation, we normalized each continuous valued feature to have zero mean and unit variance.

**Table 2:** Domain expert identified features with percent missing. Heart rate, temperature, respiratory rate, and mean arterial blood pressure differences are the difference between the most recent measurement and the average over the previous 24 hours.

Feature	Percent Missing Controls (N = 1188)	Percent Missing Culture Positive (N = 110)	Percent Missing Clinically Positive (N = 265)
Gestational age	<1%	0	<1%
Postnatal age	0	0	0
White blood cell count	74%	45%	43%
Hemoglobin	74%	45%	43%
Platelet count	74%	46%	43%
Immature to total neutrophil (I/T) ratio	75%	48%	45%
Capillary pH	95%	84%	82%
Bicarbonate	56%	33%	29%
Glucose	56%	33%	29%
Creatinine	56%	33%	29%
Respiratory rate	2%	11%	9%
Temperature	0	<1%	<1%
Heart rate	0	<1%	<1%
Systolic blood pressure	0	<1%	1%
Diastolic blood pressure	0	<1%	1%
Mean arterial pressure	9%	2%	1%
Weight	0	0	0
Fraction inspired Oxygen (FiO2)	<1%	0	0
Heart rate difference	<1%	<1%	1%
Respiratory rate difference	3%	16%	16%
Mean arterial pressure difference	39%	35%	29%
Temperature difference	12%	14%	11%

In an effort to control model overfitting, we implemented a second feature selection process as part of the hyper-parameter tuning process, referred to as “automated feature selection” in Fig 1. The automated method is based on the mutual information between each individual feature and sepsis class (case or control). Mutual information is an estimate of the dependency between two variables that quantifies the amount of information (bits) that one may infer about one variable based on the observed value of the other [44]. For each step in the outer loop of the cross-validation procedure (described below), the top *n* features were selected based on the mutual information estimate from the data in the *k-1* data folds that are used to train the model [45,46]. We employed a commonly adopted heuristic to determine *n*, in which we require at least 10 samples from each class per feature.

#### Model training

**Fig 4:**
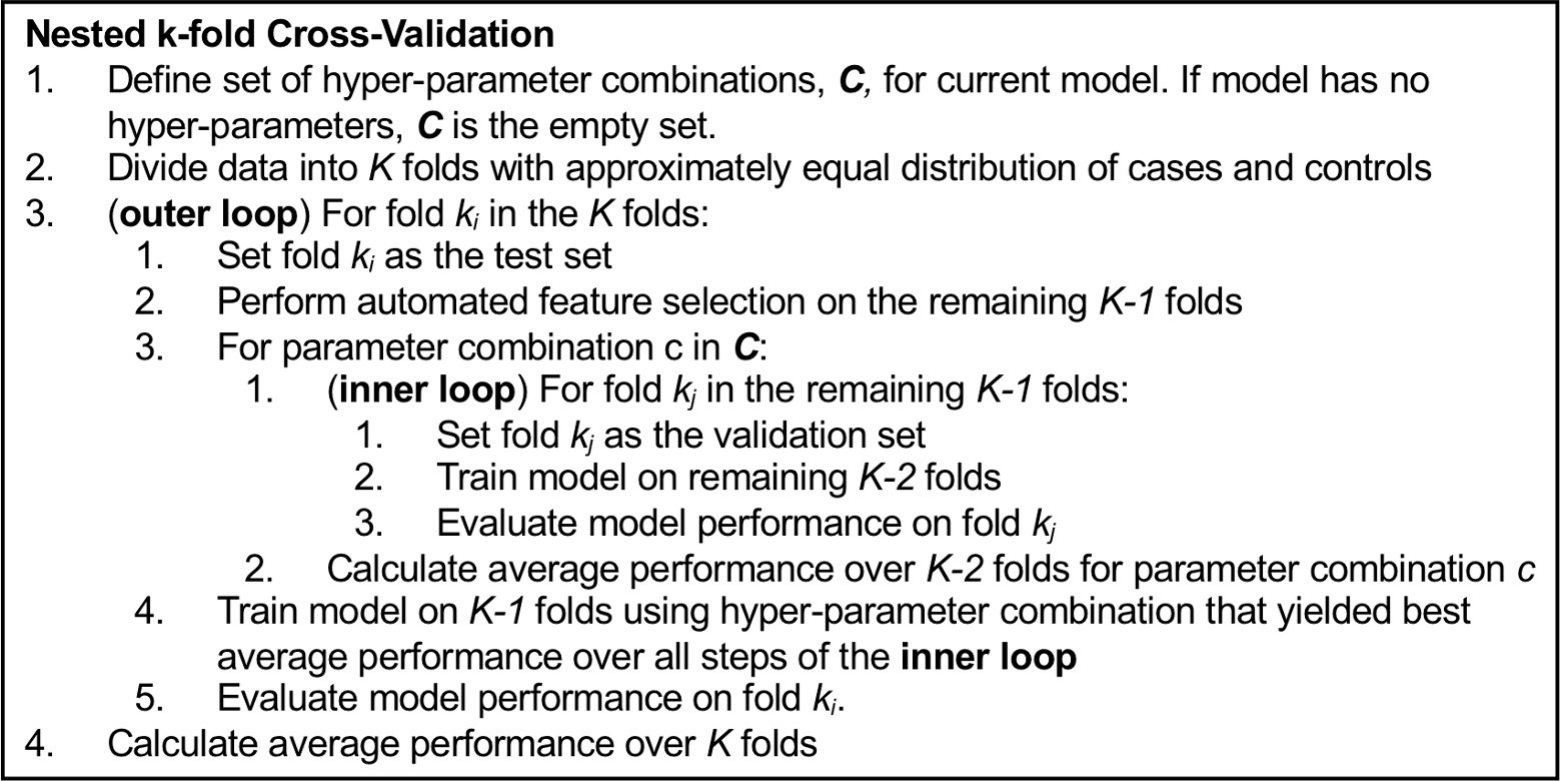
Pseudo-code for nested k-fold cross validation. The inner loop performs cross-validation to identify the best features and model hyper-parameters using the *k-1* data folds available at each iteration of the outer loop. The model is trained once for each outer loop step and evaluated on the held-out data fold. This process yields *k* evaluations of the model performance, one for each data fold, and allows the model to be tested on every sample.

We trained eight machine learning classification models to differentiate input data from control and case windows as either “sepsis negative” or “sepsis positive”: logistic regression with L2 regularization, naïve Bayes, support vector machine (SVM) with a radial basis function kernel, K-nearest neighbors (KNN), Gaussian process, random forest, AdaBoost, and gradient boosting [47–50]. As illustrated in Figs 1 and 4, we trained and evaluated each model with a nested cross-validation approach consisting of an outer evaluation loop and an inner parameter selection and training loop [51]. At initiation of the procedure, the input data set is divided into *k* folds with approximately equal numbers of cases and controls. For each iteration of the outer loop, one data fold is reserved for testing. The remaining *k-1* folds are passed to the inner loop where automated feature selection is performed followed by model parameter tuning. All models, except naïve Bayes and Gaussian process (so-called parameter-free models), include hyper-parameters that must be systematically selected independent of the evaluation data. These include, for example, regularization terms used to control potential model over-fitting to the training data, the kernel coefficient in an SVM with a radial basis function, and the number of trees in a random forest. The inner loop includes a grid search over candidate parameters. The parameter values evaluated for each model are given in Table 3. Each parameter setting is evaluated with a (*k-1)*-fold cross validation procedure (the last, *k*^*th*^, fold remains in the outer loop for evaluation). The hyper-parameters that yield the best average cross-validation area under the receiver operating characteristic (AUC) are selected. The model is then trained on the data in the *k-1* folds using the best parameters and then evaluated on its prediction performance for the held-out fold in the outer loop. This process is repeated *k* times, once for each iteration of the outer loop, resulting in *k* evaluations of model performance.

**Table 3:** Hyper-parameters and value ranges evaluated in each fold of nested cross-validation procedure. For models with more than one parameter, the cross-product of all parameter value combinations was evaluated. Detailed definitions of each parameter are available in the Python *scikit-learn* documentation (https://scikit-learn.org/stable/modules/classes.html).

Model	Parameters	Values Tested
AdaBoost	Base estimator (default values unless otherwise indicated)	Decision Tree Classifier Logistic Regression (balanced class weights) Support Vector Machine Classifier (RBF kernel, balanced class weights)
	Number of estimators	50, 100
	Learning rate	0.1, 0.5, 1.0
Gradient boosting	Number of estimators	50, 100, 200
	Individual estimator maximum depth	3, 5, 10
k-nearest neighbors	Number of neighbors	5, 10
	Neighbor weights	uniform, distance
Logistic regression	Inverse regularization	0.01, 0.1, 1, 10, 100
Random Forest	Number of estimators	10, 50, 100, 200
	Split criterion	gini, entropy
	Tree maximum depth	2, 4, 6
Support vector machine*	Inverse regularization	0.01, 0.1, 1, 10, 100
	Kernel coefficient, γ	0.01, 0.1, 1, 10, 100

*The radial basis function kernel was used for the support vector machine

#### Model evaluation

Due to the nonspecific presentation of sepsis in infants and adverse outcomes that may result from delays in therapy, physicians often rely on clinical judgment to begin empirical antibiotic therapy in infants [52]. Furthermore, it is frequently difficult to obtain an adequate volume of blood from critically ill infants, thus reducing the sensitivity of blood cultures [17,53]. As a result, cultures may be negative despite the presence of infection. Consequently, we suspect that some of the infants in the clinically positive group may be truly infected. Therefore, to gain insight on model predictive performance for cases with near certainty of sepsis and those with less certainty, we executed the model training and evaluation procedure on two data subsets, denoted *CPOnly* and *CP+Clinical*, of our overall dataset. Both subsets include all controls. However, CPOnly includes only the *culture positive* sepsis cases, whereas CP+Clinical contains the *culture positive* and the *clinically positive* cases. The prevalence of cases in the CPOnly dataset and the CP+Clinical dataset was 9% and 25.0%, respectively.

We compared inter-model performance through AUC for the CPOnly and CP+Clinical datasets. The significance of model AUC differences was evaluated by considering the mean AUC of each model over the 10 validation folds. The null hypothesis of equal inter-model AUC distributions was tested with Friedman’s rank sum test and post-hoc analysis of pairwise differences was conducted using Nemenyi’s test (R PMCMRplus, version 1.4.1).

We also compared model performance at fixed sensitivity values. All of the prediction models evaluated in this study generate a numeric score in the range 0 to 1, which can be directly interpreted as the probability of sepsis. To classify the input as positive or negative for sepsis, a numeric threshold must be selected, such that scores above that threshold are considered “sepsis positive.” To compare performance at specified sensitivity levels, we set the decision threshold independently for each model in order to achieve the desired sensitivity and report corresponding specificity, positive predictive value (PPV), and negative predictive value (NPV). For each reported performance measure, we obtain 10 observed values (one for each fold in the outer loop of the nested cross validation procedure). We report the mean value and range for each metric over the 10 observations.

We evaluated potential model overfitting (sensitivity to training data sample variance) and bias (insufficient model capacity) through learning curve analysis. A learning curve is a plot of a selected performance metric as a function of the number of training samples. Two curves are generated, one each for the training and validation sets. In the absence of bias and variance (overfitting), both the training and validation curves will approach optimum performance as the training set size increases. In the presence of variance, the training curve will approach the optimum value, but the validation curve will not. In the presence of bias, the training and validation curves will both fail to approach the optimum value.

We used the *Python scikit-learn* library [54], which contains implementations of all models used in this study, to execute all aspects of automated feature selection, hyper-parameter selection, model training, and model evaluation. Data for this study is provided in the supplementary files (S1 and S2 Files). All code is available at https://github.com/chop-dbhi/sepsis_01.

## Results

**Fig 5:**
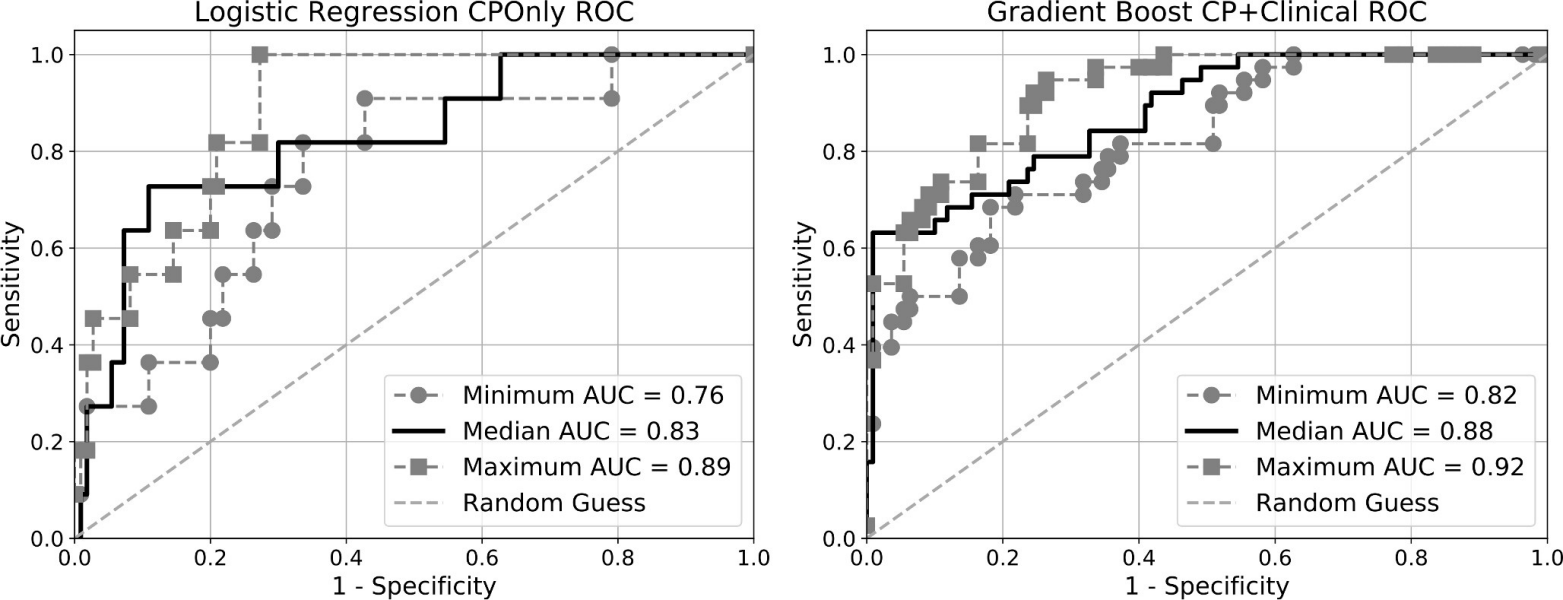
**Receiver operating characteristic (ROC) curves for the logistic regression model and the gradient boosting model on the CPOnly (left) and CP+Clinical dataset (right), respectively.** Each figure presents three curves: the solid black curve corresponds to the iteration of the nested cross-validation procedure with the median value of area under the curve (AUC), the two dashed lines represent the iterations with the minimum and maximum AUC.

We implemented eight machine learning models with the objective of identifying infant sepsis four hours prior to clinical recognition in both the CPOnly and CP+Clinical datasets. Models were tasked with classifying input data from control and case windows as either “sepsis negative” or “sepsis positive”. The AUC for all models for the CPOnly and CP+Clinical datasets is presented in Table 4 (hyper-parameters are given in S2 and S3 Tables). Representative ROC curves for models with the highest mean AUC for each dataset are presented in Fig 5. The Friedman rank sum test was used to test the null hypothesis that all models have equal AUC distributions over the 10 cross-validation folds, which was rejected for both the CPOnly and CP+Clinical dataset results with p<0.001. Post-hoc analysis with the Nemenyi test was conducted to compare differences between model pairs. For the CPOnly data set, the following statistically significant differences (p<0.05) were found: AdaBoost had higher AUC than Gaussian process and KNN; and logistic regression had a higher AUC than KNN. For the CP+Clinical dataset, the following significant differences were found: KNN had a lower AUC than gradient boosting, logistic regression, random forest, and SVM; and Gaussian process had a lower AUC than gradient boosting, logistic regression, random forest, and SVM. No statistically significant differences were found for any other pairs.

**Table 4:** Area under receiver operating characteristic for *CPOnly* (controls and culture positive cases) and *CP+Clinical* (controls, culture positive cases, and clinically positive cases) for each model. Each value is computed as the mean over 10 iterations of cross-validation. Values in brackets indicate performance range over the 10 iterations. Bold text indicates highest performance in each column. The null hypothesis of equal inter-model distributions was rejected by the Friedman rank sum test with p-values of <0.001 for both the CPOnly and CP+Clinical datasets.

Model	CPOnly	CP+Clinical
AdaBoost	**0.83** [0.76, 0.89]	0.85 [0.80, 0.90]
Gradient boosting	0.80 [0.71, 0.91]	**0.87** [0.82, 0.92]
Gaussian process	0.75 [0.67, 0.90]	0.79 [0.69, 0.88]
k-nearest neighbors	0.73 [0.66, 0.87]	0.79 [0.72, 0.83]
Logistic regression	**0.83** [0.76, 0.89]	0.85 [0.80, 0.94]
Naïve Bayes	0.81 [0.69, 0.87]	0.84 [0.79, 0.90]
Random forest	0.82 [0.73, 0.88]	0.86 [0.82, 0.91]
Support vector machine*	0.82 [0.76, 0.88]	0.86 [0.82, 0.91]

*The radial basis function kernel was used for the support vector machine

The models evaluated in this study produce a numeric output, which can be interpreted as the probability of sepsis. By default, the input is classified as sepsis positive if the estimated probability is greater than a threshold of 0.5. However, the threshold is an adjustable parameter. We set the decision threshold independently for each model in order to facilitate model comparison with uniform (i.e. fixed) sensitivity across all models. Performance at a fixed sensitivity of 80% across all models is presented in Table 5 and Table 6 for the CPOnly and CP+Clinical datasets, respectively. Performance at 90% and 95% sensitivity is presented in the S4 and S5 Tables. The SVM model performed at least as well or better than all models for all metrics on the CPOnly dataset, while no model outperformed every other model on every metric for the CP+Clinical dataset.

**Table 5:** Classifier model prediction performance on *CPOnly* (controls and culture positive cases) for fixed sensitivity ratio of 0.8. The probability of sepsis threshold was adjusted individually for each model in each cross validation run to achieve 0.8 sensitivity. Each metric value is computed as the mean over 10 iterations of cross-validation. Values in brackets indicate performance range over the 10 iterations. Bold text indicates highest performance in each column.

Model	Specificity	PPV	NPV
AdaBoost	0.71 [0.64, 0.81]	**0.23** [0.18, 0.30]	0.97 [0.97, 0.98]
Gradient boosting	0.69 [0.58, 0.91]	**0.23** [0.16, 0.47]	**0.98** [0.97, 0.99]
Gaussian process	0.53 [0.32, 0.80]	0.16 [0.11, 0.29]	0.97 [0.95, 0.98]
K-nearest neighbors	0.20 [0, 0.77]	0.13 [0.09, 0.26]	0.29 [0, 1]
Logistic regression	0.71 [0.61, 0.82]	**0.23** [0.17, 0.31]	0.97 [0.97, 0.98]
Naïve Bayes	0.69 [0.49, 0.77]	0.22 [0.14, 0.28]	**0.98** [0.96, 0.99]
Random forest	0.71 [0.62, 0.83]	**0.23** [0.19, 0.32]	**0.98** [0.97, 0.99]
Support vector machine*	**0.72** [0.63, 0.83]	**0.23** [0.18, 0.32]	**0.98** [0.97, 0.98]

PPV: positive predictive value; NPV: negative predictive value

*The radial basis function kernel was used for the support vector machine

**Table 6:** Classifier model prediction performance on *CP+Clinical* (controls, culture positive cases, and clinically positive cases) for fixed sensitivity ratio of 0.8. The probability of sepsis threshold was adjusted individually for each model in each cross validation run to achieve 0.8 sensitivity. Each metric value is computed as the mean over 10 iterations of cross-validation. Values in brackets indicate performance range over the 10 iterations. Bold text indicates highest performance in each column.

Model	Specificity	PPV	NPV
AdaBoost	0.72 [0.62, 0.82]	0.51 [0.43, 0.62]	0.92 [0.91, 0.95]
Gradient boosting	**0.74** [0.63, 0.84]	**0.53** [0.43, 0.63]	0.92 [0.91, 0.93]
Gaussian process	0.60 [0.32, 0.85]	0.44 [0.29, 0.65]	0.90 [0.83, 0.93]
K-nearest neighbors	0.55 [0.4, 0.67]	0.39 [0.32, 0.46]	0.90 [0.86, 0.92]
Logistic regression	**0.74** [0.65, 0.82]	0.52 [0.45, 0.62]	**0.93** [0.91, 0.95]
Naïve Bayes	0.73 [0.63, 0.85]	0.52 [0.42, 0.66]	0.92 [0.91, 0.93]
Random forest	**0.74** [0.56, 0.84]	**0.53** [0.39, 0.63]	0.92 [0.90, 0.93]
Support vector machine*	0.72 [0.6, 0.85]	0.51 [0.41, 0.65]	0.92 [0.90, 0.93]

PPV: positive predictive value; NPV: negative predictive value

*The radial basis function kernel was used for the support vector machine

**Fig 6:**
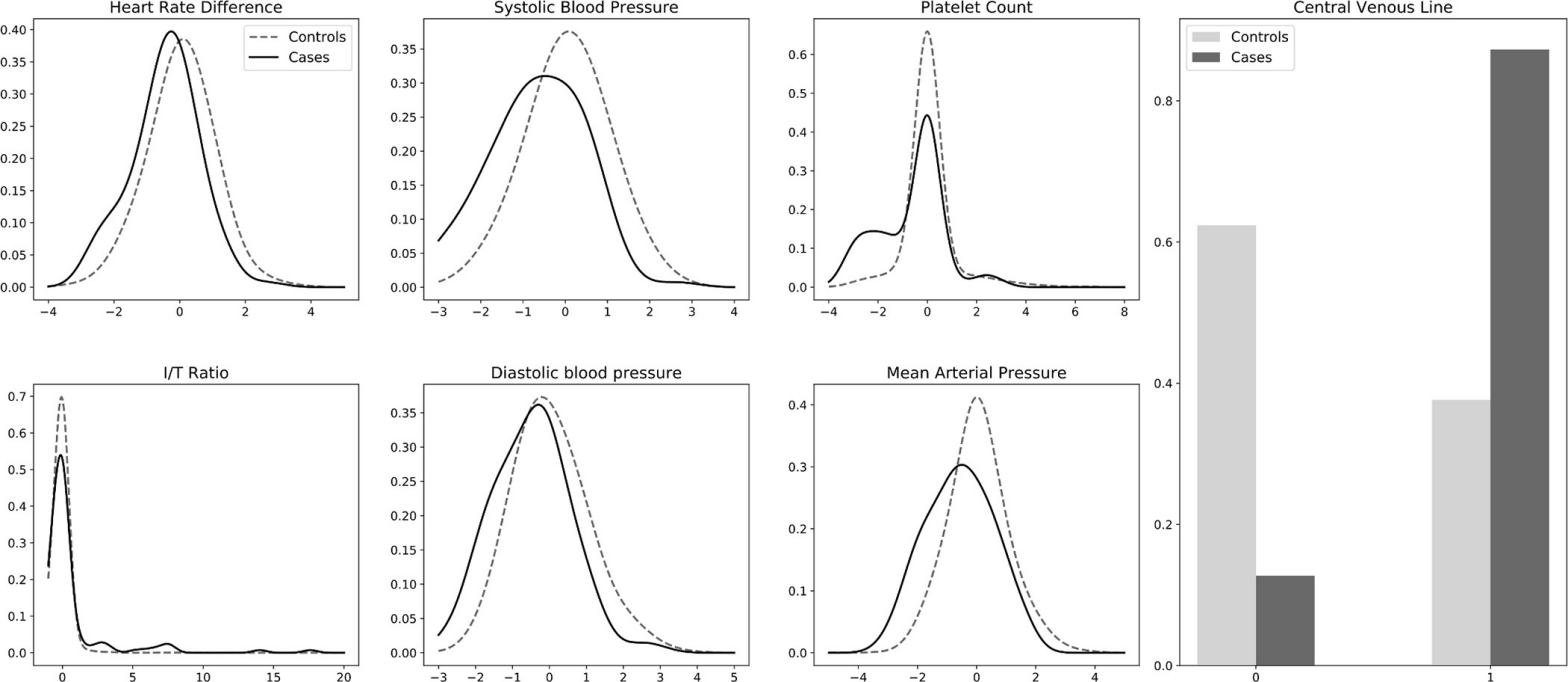
Density estimates of continuous valued and distribution of binary valued features. Only features where the mean magnitude of the logistic regression coefficient was ≥ 0.1 and the feature was selected by the univariate feature selection process more than half of the cross-validation iterations for the CPOnly dataset are shown. Dashed lines indicate controls, solid lines indicate cases. The horizontal axis indicates the normalized feature value. The vertical axis indicates the proportion of samples with the feature value.

We applied an automated univariate feature selection method using mutual information (see Fig 1) to select the top *n* features for each iteration of the nested *k*-fold procedure (see S6 Table). Based on the number of cases available in the training data, 11 features were selected for the CPOnly dataset, and 35 (i.e. all features) were selected for the CP+Clinical dataset. Different features may be selected for each iteration. To gain some insight on feature importance, we examined features selected for the CPOnly dataset for more than half of the cross-validation iterations for which the mean magnitude of the logistic regression coefficient was greater than 0.095 as shown in Table 7. The corresponding coefficients obtained for the logistic regression model, which was among the best models, are also shown. The coefficients obtained for the CP+Clinical dataset are included for comparison. Kernel density estimates for the continuous features and distributions for binary features are shown in Fig 6. The density estimates show that there are differences between the distributions of the culture positive cases and the controls. We note that diastolic blood pressure and mean arterial pressure have coefficients with different signs in the two datasets. It is possible that these differences reflect an actual difference in the distribution of those variables between the two different populations of cases. Alternatively, there may be interactions with other variables that differ between the two populations. For example, “heart rate” has a positive coefficient (not shown) combined with a negative “heart rate difference” coefficient that suggests an interaction between increased risk associated with elevated heart rate and decreased risk associated with heart rate variability that may be explained in part by known sepsis pathophysiology and prior research [55,56].

**Table 7:** Features selected by the univariate feature selection process more than half of the cross-validation iterations for the CPOnly dataset where the mean magnitude of the logistic regression coefficient was ≥ 0.1. The *CPOnly Count* column indicates the number of iterations out of ten for which the feature was selected. All features were used in every iteration for the CP+Clinical dataset. The *CPOnly Coefficient* and *CP+Clinical Coefficient* indicate the mean coefficient for the feature as learned by the logistic regression classifier for the CPOnly and CP+Clinical datasets, respectively. Positive coefficients (bold text) indicate features for which positive values are associated with an *increase* in the predicted sepsis probability. Negative coefficients (italics text) indicate features for which positive values are associated with a *decrease* in the predicted sepsis probability. The “difference” variables are positive when the value has increased compared to the patient’s average over the previous 24 hours.

	CPOnly		CP+Clinical
Feature	Count	Coefficient	Coefficient
Central venous line	10	**1.85**	**1.28**
Heart rate difference	9	*-0*.*42*	*-0*.*13*
Systolic blood pressure	10	*-0*.*38*	*-0*.*26*
Platelet count	9	*-0*.*36*	*-0*.*11*
Immature to total neutrophil (I/T) ratio	9	**0.25**	**0.16**
Diastolic blood pressure	9	**0.16**	*-0*.*06*
Mean arterial pressure	10	*-0*.*13*	**0.28**

We performed a secondary analysis to further evaluate feature importance by examining the performance of the SVM model on the CP+Clinical dataset when cumulatively removing input features. The results, shown in Table 8, suggest remarkable robustness to feature removal. There appears to be a weak interaction between the clinical attributes, which seem to lower the sensitivity, and the imputed values, which appear to improve specificity and positive predictive value (PPV), and the remaining features which appear to improve specificity without affecting sensitivity.

**Table 8:** SVM with radial basis kernel performance when removing input features. Features are removed cumulatively, that is each row represents performance when removing all features indicated in the rows up to and including the current row. Metric values are computed as the mean over 10 iterations of cross-validation. Bold text indicates best performance in each column.

Features Removed	AUC	Sensitivity	Specificity	PPV	NPV
None	**0.86**	0.74	0.79	**0.57**	**0.90**
Age, Gestational Age	0.85	0.74	**0.81**	**0.57**	**0.90**
*Missing above 50%	0.84	0.74	0.77	0.52	**0.90**
^+^Comorbidities	0.84	**0.75**	0.77	0.53	**0.90**
^x^Clinical assessments / indwelling lines / support	0.84	**0.75**	0.77	0.53	**0.90**

PPV: positive predictive value; NPV: negative predictive value

*Hemoglobin, I/T Ratio, platelet count, white blood cell count

^+^congenital heart disease, chronic lung disease, necrotizing enterocolitis, intraventricular hemorrhage/ventriculo-peritoneal shunt, other surgical conditions

^x^apnea/bradycardia/desaturation events, lethargy, poor perfusion, central venous line, umbilical artery catheter, extracorporeal membrane oxygenation, mechanical ventilation

**Fig 7:**
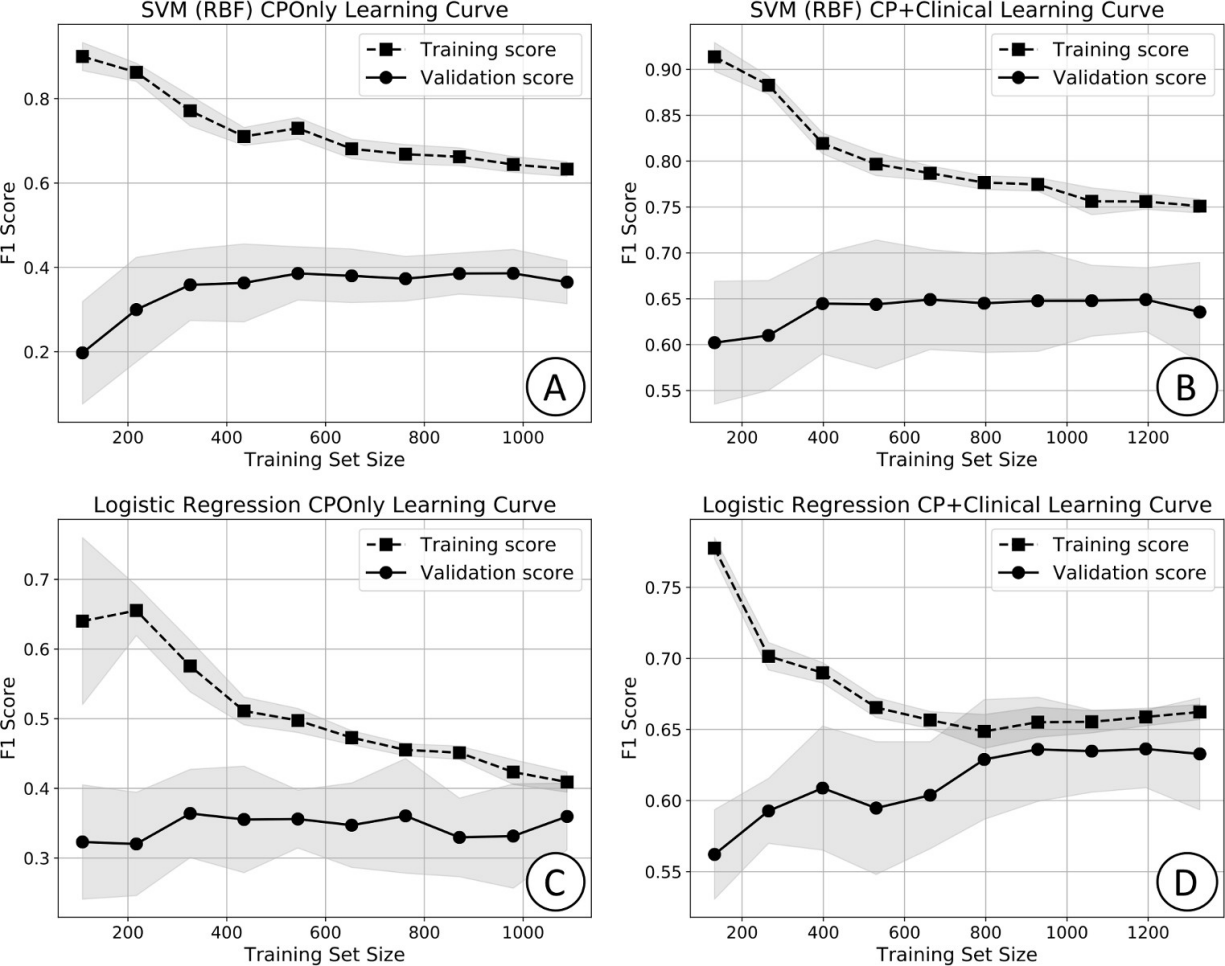
**Learning curves (LC) for:** (A) SVM on the CPOnly dataset; (B) SVM on the CP+Clinical dataset; (C) logistic regression on the CPOnly dataset; (D) logistic regression on the CP+Clinical dataset. Performance was evaluated by 10-fold cross validation. Symbols indicate the mean value over the 10 folds and the shaded region indicates one standard deviation. Optimal F1 score (y-axis) is 1.0.

Learning curves for the SVM and logistic regression models are shown in Fig 7. The y-axis indicates the F1 score (harmonic average of sensitivity and PPV) for which the optimal value is 1.0. The asymptotic difference in performance between the training and validation curves for the SVM model indicates variance is present on both datasets. A similar result was observed for all models other than the logistic regression model, which is likely due to its lower relative capacity as the only linear model. The learning curves also suggest model bias exists as indicated by failure of the training score to approach the optimal metric value. A similar result was observed for all models.

## Discussion

We found that machine learning models that utilize input features derived from data collected in most EHRs can predict sepsis in infants hospitalized in the NICU hours prior to clinical recognition. Perhaps surprisingly, we found that of the eight models considered, six (AdaBoost, gradient boosting, logistic regression, Naïve Bayes, random forest, and SVM) performed well, with no statistically significant pairwise differences in AUC, on both the CPOnly and CP+Clinical datasets. The logistic regression model was tied with AdaBoost for the highest mean AUC on the CPOnly dataset. On the CP+Clinical dataset, the logistic regression model mean AUC was only 0.02 less (not statistically significant) than that of the gradient boosting model which had the highest mean AUC. Additionally, the logistic regression model had mean PPV, NPV and sensitivity scores that were within 0.01 of the highest scores obtained by other models on both the CPOnly and CP+Clinical datasets. Importantly, learning curve analysis suggests the logistic regression model is the only model not meaningfully affected by data sample variance (i.e. overfitting). This is likely because it is a linear model whereas the five other high performing models are non-linear with greater capacity and therefore are likely more susceptible to overfitting. From this, one may conclude that, in the absence of additional training data, which could help reduce variance in the other models, the logistic regression model will best generalize to other datasets using the same input features.

As our AUC results in Table 4 indicate, most models performed nearly the same on both datasets, but some performed better on the CP+Clinical dataset as compared to the CPOnly dataset. We had anticipated that the models would perform better on the CPOnly dataset because the CP+Clinical dataset likely includes cases that were treated as sepsis by clinicians even though they may have actually been sepsis negative. Conceptually, such samples act as noise in the training data because they may be mislabeled relative to the true, unobservable, decision boundaries in the input space. In contrast, all cases in the CPOnly data set are expected to represent sepsis given their association with positive blood cultures. We hypothesize that two factors are at play. The first is that it is likely the majority of the *clinically positive* cases are indeed positive for sepsis. If so, the models in turn benefit from a decrease in class imbalance relative to the CPOnly dataset. It is well established that class imbalance generally degrades machine learning model performance and remains challenging despite development of methods designed to counter its impact [57,58]. A second factor is likely the limited number of cases available for learning in the CPOnly dataset.

The KNN model had notably poor performance compared to the other models on the CPOnly dataset. In fact, it and the Gaussian process model, were the only models to have statistically significant lower pairwise mean AUC compared to the best performing models. This is most likely attributable to the small number of cases. The KNN model is based on a voting scheme wherein the predicted class of the input is taken to be that of majority class of the input’s closest neighbors in feature space. For sparse data, particularly when one class is significantly underrepresented as is the case here, the KNN model will generally perform poorly simply because there are few available samples to populate the region of input space that correlates to the minority class (culture positive sepsis in this case).

The ROC curves in Fig 5 indicate that some tuning of the decision threshold is possible to increase specificity or sensitivity as warranted by the intended application. For example, if the prediction model is used as a screening tool, wherein avoiding false negatives is critical, the decision threshold can be lowered. There are limitations however; as indicated by Fig 5, arbitrarily increasing the sensitivity to 100% (i.e. no false negatives) can drastically decrease specificity which may not be acceptable. For example unnecessary antibiotic exposure in non-infected infants may worsen clinical outcomes and contribute to the development of antibiotic resistance [59–61]. These studies underscore the importance of developing novel, improved methods for sepsis detection in infants with potentially life-threatening illness while minimizing the overtreatment of non-infected infants. Nevertheless, due to the severity of sepsis outcomes with delays in diagnosis and treatment, particularly in infants, greater importance is placed on identifying positive cases (high sensitivity), often at the expense of assessing a large number of negative cases (low PPV). When we considered model performance for a high, fixed sensitivity value of 80%, we found that the models properly screened at least 68% of the negative cases. Although the PPV was lower (23% and 53% on our two datasets), the overall results imply that machine learning models can provide significant benefit by reducing clinician burden and cost by lowering the number of false positive cases reviewed by care providers. At the same time, the models provide early recognition of sepsis that may facilitate earlier treatment and potentially improve patient outcomes.

The few previous studies that also considered methods for early sepsis recognition are detailed in Table 9. Only one study used input features limited to those commonly found in the EHR; however it was conducted for an adult cohort [31]. Similarly, only one previous study considered an infant cohort; however it included high frequency vital sign measurements as inputs and the reported results do not include an explicit description of model sepsis prediction time relative to clinical recognition [28]. Although it is difficult to draw direct comparisons because of dataset differences, population differences, and limited reporting of outcome metrics, we did find that the best models in our study compared favorably in terms of area under the receiver operating characteristic (AUC). The best models in this study achieved a mean AUC of 0.80–0.82 and 0.85–0.87 (see Table 4) on the CPOnly and CP+Clinical datasets, respectively, which are on par with the values of 0.74 and 0.72 reported for these two prior studies. We caution, however, that because we excluded data within 10 days of any sepsis evaluation from our analysis, it is possible that our models will not perform as well in a real-world setting in which data are evaluated during these time periods. The remaining two studies were both performed in adults and included high frequency vital sign data [22,24] which are not typically available in most EHRs. Additionally, the reported results for one of these are based on a nearly balanced test set (3:2 ratio of cases to controls) and therefore may be optimistic [22]. It is well known that class imbalance is a significant challenge for machine learning models. Our results reflect very imbalanced datasets (case prevalence of 9% and 25.0% for our CPOnly and CP+Clinical datasets, respectively) that are closer to the estimated true incidence rates that a real-world model will encounter.

**Table 9:** Selected studies applying machine learning for sepsis recognition. Prediction time indicates the time of model prediction relative to time of draw for blood culture.

Paper	Population	Prediction time	Data Sources*	Method†	AUC
Desautels 2016 [31]	Adult	4-hours prior	Demographics, Clinical, Vitals	Proprietary (*InSight*)	0.74
Fairchild 2017 [28]	**Infant**	unspecified	HF Vitals§	LR	0.72
Shashikumar 2017 [22]	Adult	4-hours prior	Demographics, Clinical, HF Vitals§	SVM	0.8
Nemati 2017 [24]	Adult	4-hours prior	Demographics, HF Vitals§, Labs	Weibull-Cox PHR	0.85

*Data sources described as “clinical” typically refer to physical exam observations (e.g. perfusion status).

†Abbreviations for methods: PHR–proportional hazard ratio; SVM–support vector machine; LR–logistic regression

‡ AUC–area under receiver operating characteristic

§High-frequency (HF) vitals: Studies that included vital sign measures every 5 seconds or more frequently were designated as “high-frequency vitals.” All other studies that included vital signs typically used measurements that were recorded hourly.

A significant strength of our models is that all of the features are derived from values routinely collected in most EHRs. Further, there are several freely available software libraries that aid implementations of the machine learning methods used in our models. Once trained, the prediction models can generate new predictions for a given input quickly using only modest computational resources (e.g. a single personal computer). Given these considerations, we expect that the approach presented here is generalizable and will have the potential for adoption at many institutions. We note that the models would almost definitely require retraining for non-NICU settings, however the method should still be applicable.

There are important limitations to our current models. Most notably, the learning curves (see Fig 7) indicate the presence of variance on all models except the logistic regression model. It is possible that additional training samples may reduce model variance and improve overall performance. A general challenge in machine learning, that applies here, is the difficulty in determining the number of training examples necessary to properly fit a model. Although we continue to collect data as part of our ongoing research, it is possible that many more samples are needed than will be possible to collect in a reasonable timeframe. Model bias indicated in the learning curves also suggest that our models may be improved by adding model complexity either in the form of additional features or more complex model architectures. As part of our future research we will investigate the use of additional input features extracted from clinical notes and high frequency vital sign data as a potential approach to model improvement. An additional limitation is our use of mean imputation to address missing data, which induces bias in the relationship between variables and may not perform as well as more robust methods, such as KNN and multiple imputation. As noted in the methods section, such approaches introduce additional tuning parameters and hence the potential for increased overfitting and reduced generalizability. However, as we continue to collect more data, the risk of overfitting may decrease so that more advanced imputation methods may be considered. An additional concern about all imputation methods, including mean, is that they are based on assumptions about the manner in which data are missing (e.g. missing at random). Alternatively, Bayesian models, such as probabilistic graphical models, can typically avoid missing data issues entirely for individual predictions by summing out the missing variables in the posterior probability estimates. We intend to evaluate these methods in our continued research.

In conclusion, our results demonstrate that several machine learning methods can be used to develop models that can help identify infant sepsis hours prior to clinical recognition while screening a large portion of negative cases and may therefore be valuable as a clinical decision support tool. In this study, the logistic regression model stood out in that it had nearly equivalent performance to the highest performing model for all analyses, while being the most resilient to overfitting. Further research is warranted to assess prediction performance improvements through inclusion of additional input features. As previously reported, challenges may be expected in translating retrospective sepsis decision support models into effective clinical tools [21,33]. Therefore, further research that includes performance of a clinical trial is necessary to measure the clinical utility of machine learning models for early recognition of sepsis in infants.

## Supporting information

S1 Table Model input features by feature group. Binary indicator variables are input as zero or one indicating presence or absence of noted observation. Normalized numerical values are input after normalizing to zero mean and unit variance. Observation frequency indicates how often the variable is typically recorded in the EHR during the course of clinical care: *random*–dependent on clinician observation and event occurrence; *hourly–*recorded hourly as part of routine care*; daily or less–*occur no more than once per day; *once*–static information.
(DOCX)

S2 Table CPOnly Hyper-parameters. Selected hyper-parameters for each fold of the nested k-fold cross-validation procedure for the CPOnly (controls and culture positive cases) dataset. Detailed definitions of each parameter are available in the Python scikit-learn documentation: https://scikit-learn.org/stable/modules/classes.html.
(DOCX)

S3 Table CP+Clinical Hyper-parameters. Table C: Selected hyper-parameters for each fold of the nested k-fold cross-validation procedure for the CP+Clinical (controls, culture positive cases, and clinically positive cases) dataset. Detailed definitions of each parameter are available in the Python scikit-learn documentation: https://scikit-learn.org/stable/modules/classes.html.
(DOCX)

S4 Table CPOnly fixed sensitivity classifier performance. Classifier model prediction performance on CPOnly (controls and culture positive cases) for fixed sensitivity ratio of 0.90 and 0.95 The probability of sepsis threshold was adjusted individually for each model in each cross validation run to achieve specified sensitivity. Each metric value is computed as the mean over 10 iterations of cross-validation. Values in brackets indicate performance range.
(DOCX)

S5 Table CP+Clinical fixed sensitivity classifier performance. Classifier model prediction performance on CP+Clinical (controls, culture positive cases, and clinically positive cases) for fixed sensitivity ratio of 0.90 and 0.95. The probability of sepsis threshold was adjusted individually for each model in each cross validation run to achieve specified sensitivity. Each metric value is computed as the mean over 10 iterations of cross-validation. Values in brackets indicate performance range.
(DOCX)

S6 Table Feature selection. Selected input features for each fold of the nested k-fold cross-validation procedure for the CPOnly (controls and culture positive cases) dataset. An X in the fold column j indicates feature in corresponding feature in row i was selected by the automated feature selection process (univariate mutual information).
(DOCX)

S1 File Cases data file. CSV file containing data for 375 sepsis case samples. Each row represents one case episode. Column 1, *sepsis_group*, indicates whether the case was a culture positive evaluation (group 1) or a clinically positive evaluation (group 3).
(CSV)

S2 File Controls data file. CSV file containing data for 1,100 control samples. Each row represents one control episode.
(CSV)

## References

[1] Qazi SA, Stoll BJ. Neonatal Sepsis. Pediatr Infect Dis J [Internet]. 2009;28(Supplement):S1–2. Available from: http://content.wkhealth.com/linkback/openurl?sid=WKPTLP:landingpage&an=00006454-200901001-00001 19106756 10.1097/INF.0b013e31819587a9

[2] Liu L, Oza S, Hogan D, Perin J, Rudan I, Lawn JE, et al Global, regional, and national causes of child mortality in 2000–13, with projections to inform post-2015 priorities: an updated systematic analysis. Lancet (London, England) [Internet]. 2015 1 31 [cited 2018 Jan 26];385(9966):430–40. Available from: http://linkinghub.elsevier.com/retrieve/pii/S0140673614616986 10.1016/S0140-6736(14)61698-6 25280870

[3] Zea-Vera A, Ochoa TJ. Challenges in the diagnosis and management of neonatal sepsis. J Trop Pediatr. 2015;61(1):1–13. 10.1093/tropej/fmu079 25604489 PMC4375388

[4] Wang H, Coates MM, Coggeshall M, Dandona L, Fraser M, Fullman N, et al Global, regional, national, and selected subnational levels of stillbirths, neonatal, infant, and under-5 mortality, 1980–2015: a systematic analysis for the Global Burden of Disease Study 2015. Lancet. 2016;388(10053):1725–74. 10.1016/S0140-6736(16)31575-6 27733285 PMC5224696

[5] Stoll BJ, Hansen NI, Sanchez PJ, Faix RG, Poindexter BB, Van Meurs KP, et al Early Onset Neonatal Sepsis: The Burden of Group B Streptococcal and E. coli Disease Continues. Pediatrics [Internet]. 2011;127(5):817–26. Available from: http://pediatrics.aappublications.org/cgi/doi/10.1542/peds.2010-2217 21518717 10.1542/peds.2010-2217 PMC3081183

[6] Stoll BJ, Hansen NI, Adams-Chapman I, Fanaroff AA, Hintz SR, Vohr B, et al Neurodevelopmental and growth impairment among extremely low-birth-weight infants with neonatal infection. J Am Med Assoc. 2004;292(19):2357–65. 10.1001/jama.292.19.2357 15547163

[7] Stoll BJ, Hansen NI, Bell EF, Shankaran S, Laptook AR, Walsh MC, et al Neonatal Outcomes of Extremely Preterm Infants From the NICHD Neonatal Research Network. Pediatrics [Internet]. 2010;126(3):443–56. Available from: http://pediatrics.aappublications.org/cgi/doi/10.1542/peds.2009-2959 20732945 10.1542/peds.2009-2959 PMC2982806

[8] Cohen-Wolkowiez M, Moran C, Benjamin DK, Cotten CM, Clark RH, Benjamin DK, et al Early and late onset sepsis in late preterm infants. Pediatr Infect Dis J [Internet]. 2009 12 [cited 2018 Feb 3];28(12):1052–6. Available from: http://www.ncbi.nlm.nih.gov/pubmed/19953725 19953725 10.1097/inf.0b013e3181acf6bd PMC2798577

[9] Cohen J, Vincent JL, Adhikari NKJ, Machado FR, Angus DC, Calandra T, et al Sepsis: A roadmap for future research. Vol. 15, The Lancet Infectious Diseases. 2015 p. 581–614. 10.1016/S1473-3099(15)70112-X 25932591

[10] Wynn JL. Defining neonatal sepsis. Vol. 28, Current Opinion in Pediatrics. 2016 p. 135–40. 10.1097/MOP.0000000000000315 26766602 PMC4786443

[11] Balamuth F, Alpern ER, Abbadessa MK, Hayes K, Schast A, Lavelle J, et al Improving Recognition of Pediatric Severe Sepsis in the Emergency Department: Contributions of a Vital Sign–Based Electronic Alert and Bedside Clinician Identification. Ann Emerg Med. 2017;70(6):759–768.e2. 10.1016/j.annemergmed.2017.03.019 28583403 PMC5698118

[12] Weiss SL, Fitzgerald JC, Balamuth F, Alpern ER, Lavelle J, Chilutti M, et al Delayed antimicrobial therapy increases mortality and organ dysfunction duration in pediatric sepsis. Crit Care Med. 2014; 10.1097/CCM.0000000000000509 PMC4213742 25148597

[13] Seymour CW, Liu VX, Iwashyna TJ, Brunkhorst FM, Rea TD, Scherag A, et al Assessment of clinical criteria for sepsis for the third international consensus definitions for sepsis and septic shock (sepsis-3). JAMA—J Am Med Assoc. 2016; 10.1001/jama.2016.0288 PMC5433435 26903335

[14] Castellanos-Ortega A, Suberviola B, Garcia-Astudillo L, Holanda MS, Ortiz F, Llorca J, et al Impact of the Surviving Sepsis Campaign protocols on hospital length of stay and mortality in septic shock patients: Results of a three-year follow-up quasi-experimental study. Crit Care Med [Internet]. 2010;38(4):1036–43. Available from: http://ovidsp.ovid.com/ovidweb.cgi?T=JS&PAGE=reference&D=emed9&NEWS=N&AN=2010195658 10.1097/CCM.0b013e3181d455b6 20154597

[15] Gonsalves WI, Cornish N, Moore M, Chen A, Varman M. Effects of volume and site of blood draw on blood culture results. J Clin Microbiol. 2009;47(11):3482–5. 10.1128/JCM.02107-08 19794050 PMC2772646

[16] Connell TG, Rele M, Cowley D, Buttery JP, Curtis N. How Reliable Is a Negative Blood Culture Result? Volume of Blood Submitted for Culture in Routine Practice in a Children’s Hospital. Pediatrics [Internet]. 2007;119(5):891–6. Available from: http://pediatrics.aappublications.org/cgi/doi/10.1542/peds.2006-0440 17473088 10.1542/peds.2006-0440

[17] Schelonka RL, Chai MK, Yoder BA, Hensley D, Brockett RM, Ascher DP. Volume of blood required to detect common neonatal pathogens. J Pediatr. 1996;129(2):275–8. 8765627 10.1016/s0022-3476(96)70254-8

[18] Henry KE, Hager DN, Pronovost PJ, Saria S. A targeted real-time early warning score (TREWScore) for septic shock. Sci Transl Med [Internet]. 2015 8 5;7(299):299ra122–299ra122. Available from: http://stm.sciencemag.org/cgi/doi/10.1126/scitranslmed.aab3719 26246167 10.1126/scitranslmed.aab3719

[19] Gultepe E, Green JP, Nguyen H, Adams J, Albertson T, Tagkopoulos I. From vital signs to clinical outcomes for patients with sepsis: a machine learning basis for a clinical decision support system. J Am Med Informatics Assoc [Internet]. 2014 3 1 [cited 2018 Jan 3];21(2):315–25. Available from: https://academic.oup.com/jamia/article-lookup/doi/10.1136/amiajnl-2013-001815 10.1136/amiajnl-2013-001815 PMC3932455 23959843

[20] Kam HJ, Kim HY. Learning representations for the early detection of sepsis with deep neural networks. Comput Biol Med [Internet]. 2017 10 1 [cited 2018 Jan 8];89:248–55. Available from: https://proxy.library.upenn.edu:2067/science/article/pii/S0010482517302743 10.1016/j.compbiomed.2017.08.015 28843829

[21] Umscheid CA, Betesh J, VanZandbergen C, Hanish A, Tait G, Mikkelsen ME, et al Development, implementation, and impact of an automated early warning and response system for sepsis. J Hosp Med [Internet]. 2015 1;10(1):26–31. Available from: http://doi.wiley.com/10.1002/jhm.2259 25263548 10.1002/jhm.2259 PMC4410778

[22] Shashikumar SP, Li Q, Clifford GD, Nemati S. Multiscale network representation of physiological time series for early prediction of sepsis. Physiol Meas. 2017;38(12):2235–48. 10.1088/1361-6579/aa9772 29091053 PMC5736369

[23] Shashikumar SP, Stanley MD, Sadiq I, Li Q, Holder A, Clifford GD, et al Early sepsis detection in critical care patients using multiscale blood pressure and heart rate dynamics. J Electrocardiol [Internet]. 2017 11 1 [cited 2018 Jan 3];50(6):739–43. Available from: http://www.ncbi.nlm.nih.gov/pubmed/28916175 10.1016/j.jelectrocard.2017.08.013 28916175 PMC5696025

[24] Nemati S, Holder A, Razmi F, Stanley MD, Clifford GD, Buchman TG. An Interpretable Machine Learning Model for Accurate Prediction of Sepsis in the ICU. Crit Care Med [Internet]. 2017 12 1 [cited 2018 Jan 8];1 Available from: https://insights.ovid.com/pubmed?pmid=29286945 10.1097/CCM.0000000000002936 PMC5851825 29286945

[25] Taylor RA, Pare JR, Venkatesh AK, Mowafi H, Melnick ER, Fleischman W, et al Prediction of In-hospital Mortality in Emergency Department Patients With Sepsis: A Local Big Data-Driven, Machine Learning Approach. Jones A, editor. Acad Emerg Med [Internet]. 2016 3 1 [cited 2018 Jan 8];23(3):269–78. Available from: http://doi.wiley.com/10.1111/acem.12876 26679719 10.1111/acem.12876 PMC5884101

[26] Mayhew MB, Petersen BK, Sales AP, Greene JD, Liu VX, Wasson TS. Flexible, Cluster-Based Analysis of the Electronic Medical Record of Sepsis with Composite Mixture Models. J Biomed Inform [Internet]. 2017 12 [cited 2018 Jan 8]; Available from: http://linkinghub.elsevier.com/retrieve/pii/S1532046417302691 10.1016/j.jbi.2017.11.015 PMC6014629 29196114

[27] Lake DE, Fairchild KD, Moorman JR. Complex signals bioinformatics: evaluation of heart rate characteristics monitoring as a novel risk marker for neonatal sepsis. J Clin Monit Comput [Internet]. 2014 8 [cited 2014 Sep 29];28(4):329–39. Available from: http://www.ncbi.nlm.nih.gov/pubmed/24248424 10.1007/s10877-013-9530-x 24248424 PMC4026344

[28] Fairchild KD, Lake DE, Kattwinkel J, Moorman JR, Bateman DA, Grieve PG, et al Vital signs and their cross-correlation in sepsis and NEC: A study of 1,065 very-low-birth-weight infants in two NICUs. Pediatr Res. 2017;81(2):315–21. 10.1038/pr.2016.215 28001143 PMC5309159

[29] Mani S, Ozdas A, Aliferis C, Varol HA, Chen Q, Carnevale R, et al Medical decision support using machine learning for early detection of late-onset neonatal sepsis. J Am Med Informatics Assoc [Internet]. 2014 3;21(2):326–36. Available from: http://jamia.oxfordjournals.org/lookup/doi/10.1136/amiajnl-2013-001854 10.1136/amiajnl-2013-001854 PMC3932458 24043317

[30] Horng S, Sontag DA, Halpern Y, Jernite Y, Shapiro NI, Nathanson LA. Creating an automated trigger for sepsis clinical decision support at emergency department triage using machine learning. Groza T, editor. PLoS One [Internet]. 2017 4 6 [cited 2018 Jan 8];12(4):e0174708 Available from: http://dx.plos.org/10.1371/journal.pone.0174708 28384212 10.1371/journal.pone.0174708 PMC5383046

[31] Desautels T, Calvert J, Hoffman J, Jay M, Kerem Y, Shieh L, et al Prediction of Sepsis in the Intensive Care Unit With Minimal Electronic Health Record Data: A Machine Learning Approach. JMIR Med informatics [Internet]. 2016 9 30 [cited 2018 Jan 2];4(3):e28 Available from: https://medinform.jmir.org/2016/3/e28/10.2196/medinform.5909 PMC5065680 27694098

[32] Calvert JS, Price DA, Chettipally UK, Barton CW, Feldman MD, Hoffman JL, et al A computational approach to early sepsis detection. Comput Biol Med [Internet]. 2016 7 1 [cited 2018 Jan 16];74:69–73. Available from: http://www.sciencedirect.com/science/article/pii/S0010482516301123 10.1016/j.compbiomed.2016.05.003 27208704

[33] Coggins SA, Weitkamp JH, Grunwald L, Stark AR, Reese J, Walsh W, et al Heart rate characteristic index monitoring for bloodstream infection in an NICU: A 3-year experience. Arch Dis Child Fetal Neonatal Ed. 2016;101(4):F329–32. 10.1136/archdischild-2015-309210 26518312 PMC4851911

[34] Rowley AH, Wald ER. The Incubation Period Necessary for Detection of Bacteremia in Immunocompetent Children with Fever. Clin Pediatr (Phila) [Internet]. 1986 10 2 [cited 2018 Dec 5];25(10):485–9. Available from: http://journals.sagepub.com/doi/10.1177/000992288602501001 3757393 10.1177/000992288602501001

[35] Kurlat I, Stoll BJ, McGowan JE. Time to positivity for detection of bacteremia in neonates. J Clin Microbiol [Internet]. 1989 5 1 [cited 2018 Dec 5];27(5):1068–71. Available from: http://www.ncbi.nlm.nih.gov/pubmed/2745679 2745679 10.1128/jcm.27.5.1068-1071.1989 PMC267484

[36] Padula MA, Dewan ML, Shah SS, Padula AM, Srinivasan L, McGowan KL, et al Risk Factors Associated With Laboratory-confirmed Bloodstream Infections in a Tertiary Neonatal Intensive Care Unit. Pediatr Infect Dis J [Internet]. 2014 10;33(10):1027–32. Available from: http://content.wkhealth.com/linkback/openurl?sid=WKPTLP:landingpage&an=00006454-201410000-00009 10.1097/INF.0000000000000386 24776516

[37] Verstraete EH, Blot K, Mahieu L, Vogelaers D, Blot S. Prediction models for neonatal health care-associated sepsis: a meta-analysis. Pediatrics [Internet]. 2015 4 1 [cited 2018 Apr 23];135(4):e1002–14. Available from: http://www.ncbi.nlm.nih.gov/pubmed/25755236 10.1542/peds.2014-3226 25755236

[38] Wang HE, Shapiro NI, Griffin R, Safford MM, Judd S, Howard G. Chronic Medical Conditions and Risk of Sepsis. Gold JA, editor. PLoS One [Internet]. 2012 10 31 [cited 2018 May 18];7(10):e48307 Available from: http://dx.plos.org/10.1371/journal.pone.0048307 23118977 10.1371/journal.pone.0048307 PMC3485139

[39] Yende S, Iwashyna TJ, Angus DC. Interplay between sepsis and chronic health. Trends Mol Med [Internet]. 2014 4 1 [cited 2018 May 18];20(4):234–8. Available from: https://proxy.library.upenn.edu:2067/science/article/pii/S147149141400029X?via%3Dihub 10.1016/j.molmed.2014.02.005 24636941 PMC4016696

[40] Odetola FO, Gebremariam A, Freed GL. Patient and Hospital Correlates of Clinical Outcomes and Resource Utilization in Severe Pediatric Sepsis. Pediatrics [Internet]. 2007 3 1;119(3):487–94. Available from: http://pediatrics.aappublications.org/cgi/doi/10.1542/peds.2006-2353 17332201 10.1542/peds.2006-2353

[41] Beretta L, Santaniello A. Nearest neighbor imputation algorithms: a critical evaluation. BMC Med Inform Decis Mak [Internet]. 2016 [cited 2018 Dec 12];16 Suppl 3(Suppl 3):74 Available from: http://www.ncbi.nlm.nih.gov/pubmed/27454392 27454392 10.1186/s12911-016-0318-z PMC4959387

[42] Dong Y, Peng C-YJ. Principled missing data methods for researchers. Springerplus [Internet]. 2013 12 [cited 2018 Dec 12];2(1):222 Available from: http://www.ncbi.nlm.nih.gov/pubmed/23853744 10.1186/2193-1801-2-222 23853744 PMC3701793

[43] Musil CM, Warner CB, Yobas PK, Jones SL. A Comparison of Imputation Techniques for Handling Missing Data. West J Nurs Res [Internet]. 2002 11 1 [cited 2018 Dec 12];24(7):815–29. Available from: http://journals.sagepub.com/doi/10.1177/019394502762477004 12428897 10.1177/019394502762477004

[44] Kraskov A, Stögbauer H, Grassberger P. Estimating mutual information. Phys Rev E [Internet]. 2004 6 23 [cited 2018 Dec 13];69(6):066138 Available from: https://link.aps.org/doi/10.1103/PhysRevE.69.066138 10.1103/PhysRevE.69.066138 15244698

[45] Guyon I, Elisseeff A. An Introduction to Variable and Feature Selection. J Mach Learn Res [Internet]. 2003 [cited 2017 Mar 24];3(Mar):1157–82. Available from: http://www.jmlr.org/papers/v3/guyon03a.html

[46] Chandrashekar G, Sahin F. A survey on feature selection methods. Comput Electr Eng [Internet]. 2014 1 [cited 2017 Mar 24];40(1):16–28. Available from: http://linkinghub.elsevier.com/retrieve/pii/S0045790613003066

[47] Friedman J, Hastie T, Tibshirani R. The elements of statistical learning. Vol. 1 Springer series in statistics New York; 2001.

[48] Barber D. Bayesian Reasoning and Machine Learning. New York, New York, USA: Cambridge University Press; 2012 451–452 p.

[49] Hastie T, Rosset S, Zhu J, Zou H. Multi-class AdaBoost. Stat Interface [Internet]. 2009 [cited 2018 Apr 6];2(3):349–60. Available from: http://www.intlpress.com/site/pub/pages/journals/items/sii/content/vols/0002/0003/a008/

[50] Friedman JH. Stochastic gradient boosting. Comput Stat Data Anal [Internet]. 2002 2 28 [cited 2018 Apr 6];38(4):367–78. Available from: https://proxy.library.upenn.edu:2067/science/article/pii/S0167947301000652#BIB4

[51] Mani S, Ozdas A, Aliferis C, Varol HA, Chen Q, Carnevale R, et al Medical decision support using machine learning for early detection of late-onset neonatal sepsis. J Am Med Informatics Assoc [Internet]. 2014 3 1 [cited 2014 Mar 24];21(2):326–36. Available from: http://www.ncbi.nlm.nih.gov/pubmed/24043317 10.1136/amiajnl-2013-001854 PMC3932458 24043317

[52] Klingenberg C, Kornelisse RF, Buonocore G, Maier RF, Stocker M. Culture-Negative Early-Onset Neonatal Sepsis—At the Crossroad Between Efficient Sepsis Care and Antimicrobial Stewardship. Front Pediatr [Internet]. 2018 [cited 2018 Dec 13];6:285 Available from: http://www.ncbi.nlm.nih.gov/pubmed/30356671 10.3389/fped.2018.00285 30356671 PMC6189301

[53] Squire E, Favara B, Todd J. Diagnosis of neonatal bacterial infection: Hematologic and pathologic findings in fatal and nonfatal cases. Obstet Gynecol Surv. 1980;35(7):448–50. 450562

[54] Pedregosa F, Varoquaux G, Gramfort A, Michel V, Thirion B, Grisel O, et al Scikit-learn: Machine Learning in {P}ython. J Mach Learn Res. 2011;12:2825–30.

[55] Fairchild KD, O’Shea TM. Heart Rate Characteristics: Physiomarkers for Detection of Late-Onset Neonatal Sepsis [Internet]. Vol. 37, Clinics in Perinatology. Elsevier; 2010 [cited 2018 Apr 23]. p. 581–98. Available from: https://proxy.library.upenn.edu:2067/science/article/pii/S009551081000076X?via%3Dihub 10.1016/j.clp.2010.06.002 20813272 PMC2933427

[56] Bohanon FJ, Mrazek AA, Shabana MT, Mims S, Radhakrishnan GL, Kramer GC, et al Heart rate variability analysis is more sensitive at identifying neonatal sepsis than conventional vital signs. Am J Surg [Internet]. 2015 10 1 [cited 2018 Apr 23];210(4):661–7. Available from: https://proxy.library.upenn.edu:2067/science/article/pii/S0002961015003505?via%3Dihub 10.1016/j.amjsurg.2015.06.002 26212391 PMC4575854

[57] He Haibo, Garcia EA. Learning from Imbalanced Data. IEEE Trans Knowl Data Eng [Internet]. 2009 9;21(9):1263–84. Available from: http://ieeexplore.ieee.org/document/5128907/

[58] Batista GEAPA Prati RC, Monard MC. A study of the behavior of several methods for balancing machine learning training data. ACM SIGKDD Explor Newsl [Internet]. 2004 6 1;6(1):20 Available from: http://portal.acm.org/citation.cfm?doid=1007730.1007735

[59] Ting JY, Synnes A, Roberts A, Deshpandey A, Dow K, Yoon EW, et al Association between antibiotic use and neonatal mortality and morbidities in very low-birth-weight infants without culture-proven sepsis or necrotizing enterocolitis. JAMA Pediatr [Internet]. 2016 12 1 [cited 2018 Apr 30];170(12):1181–7. Available from: http://archpedi.jamanetwork.com/article.aspx?doi=10.1001/jamapediatrics.2016.2132 27775765 10.1001/jamapediatrics.2016.2132

[60] Cotten CM, Taylor S, Stoll B, Goldberg RN, Hansen NI, Sanchez PJ, et al Prolonged Duration of Initial Empirical Antibiotic Treatment Is Associated With Increased Rates of Necrotizing Enterocolitis and Death for Extremely Low Birth Weight Infants. Pediatrics [Internet]. 2009;123(1):58–66. Available from: http://pediatrics.aappublications.org/cgi/doi/10.1542/peds.2007-3423 19117861 10.1542/peds.2007-3423 PMC2760222

[61] Kuppala VS, Meinzen-Derr J, Morrow AL, Schibler KR. Prolonged Initial Empirical Antibiotic Treatment is Associated with Adverse Outcomes in Premature Infants. J Pediatr [Internet]. 2011 11 1 [cited 2018 Apr 30];159(5):720–5. Available from: http://www.ncbi.nlm.nih.gov/pubmed/21784435 10.1016/j.jpeds.2011.05.033 21784435 PMC3193552

