# Supplementary material for: Machine learning models for early sepsis recognition in the neonatal intensive care unit using readily available electronic health record data
Source: PLoS One. 2019 Feb 22;14(2):e0212665. doi: 10.1371/journal.pone.0212665 (PMC6386402; doi:10.1371/journal.pone.0212665)
Supplement: S1 Table — Binary indicator variables are input as zero or one indicating presence or absence of noted observation. Normalized numerical values are input after normalizing to zero mean and unit variance. Observation frequency indicates how often the variable is typically recorded in the EHR during the course of clinical care: random–dependent on clinician observation and event occurrence; hourly–recorded hourly as part of routine care; daily or less–occur no more than once per day; once–static information. (DOCX) [file pone.0212665.s001.docx]

**S1 Table**: **Model input features by feature group**. Binary indicator variables are input as zero or one indicating presence or absence of noted observation. Normalized numerical values are input after normalizing to zero mean and unit variance. Observation frequency indicates how often the variable is typically recorded in the EHR during the course of clinical care: *random* – dependent on clinician observation and event occurrence; *hourly –* recorded hourly as part of routine care*; daily or less* – occur no more than once per day; *once* – static information.

| **Feature Group** | **Feature Name** | **Observation Frequency** |
| --- | --- | --- |
| *Clinical assessments*  Binary indicator variables | Apnea, bradycardia, other desaturation events | Random |
|  | Lethargy |  |
|  | Poor perfusion |  |
|  | |  |
| *Comorbidities*  Binary indicator variables | Chronic lung disease | Random |
|  | Congenital heart disease |  |
|  | Intraventricular hemorrhage, ventriculo-peritoneal shunt |  |
|  | Necrotizing enterocolitis |  |
|  | Other surgical conditions |  |
|  | |  |
| *Indwelling Lines*  Binary indicator variables | Central venous line | Random |
|  | Umbilical artery catheter |  |
|  | |  |
| *Laboratory Values*  Normalized numerical values | Bicarbonate | Daily or less |
|  | Capillary pH |  |
|  | Creatinine |  |
|  | Glucose |  |
|  | Hemoglobin |  |
|  | Immature to total neutrophil (I/T) ratio |  |
|  | Platelet count |  |
|  | White blood cell count |  |
|  | |  |
| *Support*  Binary indicator variables | Extracorporeal membrane oxygenation cannula | Random |
|  | Mechanical ventilation |  |
|  | |  |
| *Vital Signs*  Normalized numerical values | Age (gestational) | Once |
|  | Age (postnatal) |  |
|  | Diastolic blood pressure | Hourly |
|  | Fraction inspired Oxygen (FiO2) |  |
|  | Heart rate |  |
|  | Mean arterial pressure |  |
|  | Respiratory rate |  |
|  | Systolic blood pressure |  |
|  | Temperature |  |
|  | Weight | Daily |
|  | |  |
| *Vital Sign Differences*  Normalized numerical values | Heart rate difference | Hourly |
|  | Mean arterial pressure difference |  |
|  | Respiratory rate difference |  |
|  | Temperature difference |  |
|  | |  |
| *Vital Sign Thresholds*  Binary indicator variables | Fraction inspired Oxygen (FiO2) threshold | Hourly |
|  | Temperature threshold |  |
