# Supplementary material for: Machine learning models for early sepsis recognition in the neonatal intensive care unit using readily available electronic health record data
Source: PLoS One. 2019 Feb 22;14(2):e0212665. doi: 10.1371/journal.pone.0212665 (PMC6386402; doi:10.1371/journal.pone.0212665)
Supplement: S3 Table — Table C: Selected hyper-parameters for each fold of the nested k-fold cross-validation procedure for the CP+Clinical (controls, culture positive cases, and clinically positive cases) dataset. Detailed definitions of each parameter are available in the Python scikit-learn documentation: https://scikit-learn.org/stable/modules/classes.html. (DOCX) [file pone.0212665.s003.docx]

**S3 Table**: **CP+Clinical Hyper-parameters.** Selected hyper-parameters for each fold of the nested k-fold cross-validation procedure for the ***CP+Clinical*** (controls, culture positive cases, and clinically positive cases) dataset. Detailed definitions of each parameter are available in the Python scikit-learn documentation: <https://scikit-learn.org/stable/modules/classes.html>.

|  |  | *Fold* | | | | | | | | | |
| --- | --- | --- | --- | --- | --- | --- | --- | --- | --- | --- | --- |
| **Model** | **Parameter** | *1* | *2* | *3* | *4* | *5* | *6* | *7* | *8* | *9* | *10* |
| AdaBoost | Base estimator^+^ | LR | LR | LR | LR | LR | LR | LR | LR | LR | LR |
|  | Number of estimators | 100 | 50 | 100 | 100 | 100 | 100 | 100 | 100 | 100 | 100 |
|  | Learning rate | 1.0 | 1.0 | 1.0 | 1.0 | 1.0 | 1.0 | 1.0 | 1.0 | 1.0 | 1.0 |
| Gradient boosting | Number of estimators | 50 | 50 | 50 | 50 | 100 | 50 | 50 | 50 | 50 | 50 |
|  | Individual estimator maximum depth | 3 | 3 | 3 | 3 | 3 | 3 | 3 | 3 | 3 | 3 |
| k-nearest neighbors | Number of neighbors | 10 | 10 | 10 | 10 | 10 | 10 | 10 | 10 | 10 | 10 |
|  | Neighbor weights^x^ | D | U | D | D | D | D | D | D | D | D |
| Logistic regression | Inverse regularization | 1 | 10 | 1 | 10 | 10 | 1 | 10 | 10 | 1 | 10 |
| Random Forest | Number of estimators | 200 | 200 | 200 | 200 | 200 | 200 | 50 | 200 | 200 | 200 |
|  | Split criterion^^^ | E | G | E | E | E | G | E | G | E | G |
|  | Tree maximum depth | 6 | 6 | 6 | 6 | 6 | 6 | 6 | 6 | 6 | 6 |
| Support vector machine* | Inverse regularization | 1 | 1 | 1 | 1 | 1 | 1 | 1 | 1 | 1 | 1 |
|  | Kernel coefficient, γ | 0.01 | 0.01 | 0.01 | 0.01 | 0.01 | 0.01 | 0.01 | 0.01 | 0.01 | 0.01 |

*The radial basis function kernel was used for the support vector machine

^+^LR – Logistic Regression Classifier with default parameters

^x^D – inverse distance neighbor weights

^^^G – Gini impurity, E – Information gain entropy
