# Supplementary material for: Machine learning models for early sepsis recognition in the neonatal intensive care unit using readily available electronic health record data
Source: PLoS One. 2019 Feb 22;14(2):e0212665. doi: 10.1371/journal.pone.0212665 (PMC6386402; doi:10.1371/journal.pone.0212665)
Supplement: S5 Table — Classifier model prediction performance on CP+Clinical (controls, culture positive cases, and clinically positive cases) for fixed sensitivity ratio of 0.90 and 0.95. The probability of sepsis threshold was adjusted individually for each model in each cross validation run to achieve specified sensitivity. Each metric value is computed as the mean over 10 iterations of cross-validation. Values in brackets indicate performance range. (DOCX) [file pone.0212665.s005.docx]

**S5 Table: CP+Clinical Fixed Sensitivity Classifier Performance** Classifier model prediction performance on ***CP+Clinical*** (controls, culture positive cases, and clinically positive cases) for **fixed sensitivity ratio of 0.90 and 0.95**. The probability of sepsis threshold was adjusted individually for each model in each cross validation run to achieve specified sensitivity. Each metric value is computed as the mean over 10 iterations of cross-validation. Values in brackets indicate performance range.

| **Model** | **Specificity** | | **PPV** | | **NPV** | |
| --- | --- | --- | --- | --- | --- | --- |
|  | **90%**  **Sensitivity** | **95%**  **Sensitivity** | **90%**  **Sensitivity** | **95%**  **Sensitivity** | **90%**  **Sensitivity** | **95%**  **Sensitivity** |
| AdaBoost | 0.57 [0.33, 0.8] | 0.42 [0.15, 0.63] | 0.44 [0.32, 0.61] | 0.37 [0.28, 0.47] | 0.95 [0.92, 0.97] | 0.98 [0.94, 0.99] |
| Gradient boosting | 0.60 [0.48, 0.75] | 0.48 [0.25, 0.66] | 0.45 [0.38, 0.56] | 0.40 [0.31, 0.50] | 0.96 [0.95, 0.97] | 0.98 [0.97, 0.99] |
| Gaussian process | 0.38 [0.22, 0.55] | 0.24 [0.09, 0.41] | 0.34 [0.28, 0.41] | 0.31 [0.27, 0.36] | 0.93 [0.89, 0.96] | 0.96 [0.91, 1] |
| K-nearest neighbors | 0.20 [0, 0.54] | 0.05 [0, 0.45] | 0.31 [0.25, 0.41] | 0.27 [0.25, 0.38] | 0.38 [0, 0.95] | 0.10 [0, 0.98] |
| Logistic regression | 0.58 [0.39, 0.76] | 0.44 [0.22, 0.66] | 0.44 [0.34, 0.57] | 0.38 [0.30, 0.49] | 0.96 [0.94, 0.98] | 0.98 [0.96, 0.99] |
| Naïve Bayes | 0.55 [0.39, 0.84] | 0.43 [0.21, 0.77] | 0.42 [0.34, 0.66] | 0.38 [0.29, 0.60] | 0.95 [0.93, 0.97] | 0.98 [0.96, 0.99] |
| Random forest | 0.57 [0.45, 0.78] | 0.46 [0.22, 0.67] | 0.43 [0.36, 0.59] | 0.39 [0.30, 0.51] | 0.96 [0.94, 0.97] | 0.98 [0.96, 0.99] |
| Support vector machine* | 0.58 [0.38, 0.75] | 0.43 [0.25, 0.64] | 0.43 [0.33, 0.56] | 0.37 [0.31, 0.47] | 0.96 [0.93, 0.98] | 0.98 [0.97, 0.99] |

PPV: positive predictive value; NPV: negative predictive value

*The radial basis function kernel was used for the support vector machine
