# Supplementary material for: Machine learning models for early sepsis recognition in the neonatal intensive care unit using readily available electronic health record data
Source: PLoS One. 2019 Feb 22;14(2):e0212665. doi: 10.1371/journal.pone.0212665 (PMC6386402; doi:10.1371/journal.pone.0212665)
Supplement: S6 Table — Selected input features for each fold of the nested k-fold cross-validation procedure for the CPOnly (controls and culture positive cases) dataset. An X in the fold column j indicates feature in corresponding feature in row i was selected by the automated feature selection process (univariate mutual information). (DOCX) [file pone.0212665.s006.docx]

**S6 Table: Feature selection.** Selected input features for each fold of the nested k-fold cross-validation procedure for the ***CPOnly*** (controls and culture positive cases) dataset. An *X* in the fold column *j* indicates feature in corresponding feature in row *i* was selected by the automated feature selection process (univariate mutual information).

|  | *Fold* | | | | | | | | | |  |
| --- | --- | --- | --- | --- | --- | --- | --- | --- | --- | --- | --- |
| **Feature** | *1* | 2 | 3 | 4 | 5 | 6 | 7 | 8 | 9 | 10 | **Total** |
| Central venous line | X | X | X | X | X | X | X | X | X | X | 10 |
| Mean arterial pressure | X | X | X | X | X | X | X | X | X | X | 10 |
| Respiratory rate difference | X | X | X | X | X | X | X | X | X | X | 10 |
| Systolic blood pressure | X | X | X | X | X | X | X | X | X | X | 10 |
| Platelet count | X | X | X | X | X | X | X |  | X | X | 9 |
| Heart rate difference | X |  | X | X | X | X | X | X | X | X | 9 |
| Immature to total neutrophil (I/T) ratio | X |  | X | X | X | X | X | X | X | X | 9 |
| Diastolic blood pressure | X | X |  | X | X | X | X | X | X | X | 9 |
| Creatinine |  |  | X | X |  | X | X | X | X | X | 7 |
| Bicarbonate |  |  |  |  | X |  | X | X | X | X | 5 |
| Fraction inspired Oxygen (FiO2) | X | X | X | X | X |  |  |  |  |  | 5 |
| Age (postnatal) | X | X |  | X |  |  |  | X |  |  | 4 |
| Hemoglobin | X |  | X |  |  |  |  | X |  |  | 3 |
| Temperature |  | X |  |  |  |  | X |  | X |  | 3 |
| White blood cell count |  | X | X |  |  |  |  |  |  |  | 2 |
| Heart rate |  | X |  |  |  | X |  |  |  |  | 2 |
| Mechanical ventilation |  |  |  |  | X |  |  |  |  | X | 2 |
| Apnea |  |  |  |  |  | X |  |  |  |  | 1 |
